# Engineering high Zn in tomato shoots through expression of *AtHMA4* involves tissue-specific modification of endogenous genes

**DOI:** 10.1186/s12864-016-2990-x

**Published:** 2016-08-12

**Authors:** Maria Kendziorek, Maria Klimecka, Anna Barabasz, Sören Borg, Justyna Rudzka, Paweł Szczęsny, Danuta Maria Antosiewicz

**Affiliations:** 1 Faculty of Biology, Institute of Experimental Plant Biology and Biotechnology, Department of Plant Anatomy and Cytology, University of Warsaw, Miecznikowa str 1, 02-096 Warszawa, Poland; 2 Department. of Molecular Biology and Genetics, Aarhus University, Forsøgsvej 1, 4200 Slagelse, Denmark; 3 Institute of Biochemistry and Biophysics PAS, Pawińskiego 5a, 02-106 Warszawa, Poland; 4 Present address: Plant Biochemistry Department, Institute of Biochemistry and Biophysics PAS, Pawińskiego 5a, 02-106 Warszawa, Poland

**Keywords:** AtHMA4, Tomato, Zinc, Cadmium, Laser microdissection, Microarray

## Abstract

**Background:**

To increase the Zn level in shoots, *AtHMA4* was ectopically expressed in tomato under the constitutive CaMV 35S promoter. However, the Zn concentration in the shoots of transgenic plants failed to increase at all tested Zn levels in the medium. Modification of Zn root/shoot distribution in tomato expressing 35S::*AtHMA4* depended on the concentration of Zn in the medium, thus indicating involvement of unknown endogenous metal-homeostasis mechanisms. To determine these mechanisms, those metal-homeostasis genes that were expressed differently in transgenic and wild-type plants were identified by microarray and RT-qPCR analysis using laser-assisted microdissected RNA isolated from two root sectors: (epidermis + cortex and stele), and leaf sectors (upper epidermis + palisade parenchyma and lower epidermis + spongy parenchyma).

**Results:**

Zn-supply-dependent modification of Zn root/shoot distribution in AtHMA4-tomato (increase at 5 μM Zn, no change at 0.5 μM Zn) involved tissue-specific, distinct from that in the wild type, expression of tomato endogenous genes. First, it is suggested that an ethylene-dependent pathway underlies the detected changes in Zn root/shoot partitioning, as it was induced in transgenic plants in a distinct way depending on Zn exposure. Upon exposure to 5 or 0.5 μM Zn, in the epidermis + cortex of the transgenics’ roots the expression of the Strategy I Fe-uptake system (ethylene-dependent *LeIRT1* and *LeFER*) was respectively lower or higher than in the wild type and was accompanied by respectively lower or higher expression of the identified ethylene genes (*LeNR*, *LeACO4, LeACO5*) and of *LeChln*. Second, the contribution of *LeNRAMP2* expression in the stele is shown to be distinct for wild-type and transgenic plants at both Zn exposures. Ethylene was also suggested as an important factor in a pathway induced in the leaves of transgenic plants by high Zn in the apoplast, which results in the initiation of loading of the excess Zn into the mesophyll of “Zn accumulating cells”.

**Conclusions:**

In transgenic tomato plants, the export activity of ectopically expressed *AtHMA4* changes the cellular Zn status, which induces coordinated tissue-specific responses of endogenous ethylene-related genes and metal transporters. These changes constitute an important mechanism involved in the generation of the metal-related phenotype of transgenic tomato expressing *AtHMA4*.

**Electronic supplementary material:**

The online version of this article (doi:10.1186/s12864-016-2990-x) contains supplementary material, which is available to authorized users.

## Background

Biofortification of crop plants in Zn is frequently considered a solution to the problem of low-mineral dietary intake. Molecular approaches to this end include overexpression of metal transport genes to generate plants that more effectively translocate the micronutrient to shoots. The major processes in roots contributing to the regulation of Zn transfer to shoots include (i) in the epidermis and cortex: regulation of metal uptake efficiency; compartmentalization in roots, primarily in vacuoles, which contributes to radial transport, (ii) in the stele: regulation of the efficiency of loading the metal into xylem vessels [1]. Therefore, efforts to biofortify a target species with Zn frequently encompass expression of the metal transporters involved in these processes.

HMA4 (a P_1B_-ATPase) from *A. thaliana* and *A. halleri* participates in Zn/Cd xylem loading, thus in the control of Zn/Cd translocation to shoots [2–7]. High *HMA4* expression resulting from tandem triplication is a key element responsible for Zn/Cd hyperaccumulation in *A. halleri* [6, 8, 9]. To enhance the efficiency of translocating Zn to shoots in transformants of non-accumulating species, *HMA4* has been expressed either under the constitutive CaMV 35S promoter or under the native promoter from *A. halleri* [6, 10–13]. Zn root-to-shoot translocation and its concentration in shoots were higher in these transformants than in the wild-type. The rise in the shoot Zn content depended, however, on the Zn concentration in the medium, thus, the increase did not occur at all tested Zn levels [10–13]. It is thought that the phenomenon of the metal-supply-dependent metal accumulation detected in transgenic plants (which differs from the pattern in the wild-type) results from different transcription profiles (molecular backgrounds) of a given plant species grown at varying metal conditions (low, sufficient, excess), against which the expression of a transgene takes place [14]. The nature of these interactions is very poorly understood.

Overall, this study contributes to a better understanding of the mechanisms underlying generation of a transgenic phenotype. The first aim of our research was to identify the metal homeostasis genes in *AtHMA4-*expressing tomato plants that were differently expressed in selected root tissues compared with wild-type plants and whose expression accompanied Zn supply-dependent changes of Zn root/shoot distribution. Laser Capture Microdissection (LCM) was used to isolate two root sectors having different functions in the control of metal root-to-shoot translocation, the (i) epidermis + cortex and (ii) stele; next, they were used for comparative expression analysis. Moreover, since expression of *AtHMA4* in tobacco and tomato leaves leads to development of necrotic areas that begin to form in the palisade parenchyma [15], two leaf sectors, (i) upper epidermis + palisade parenchyma and (ii) lower epidermis + spongy parenchyma, were also subjected to comparative expression analysis. The aim of the second part of the study was to identify metal-homeostasis pathways modified due to transgene expression accompanying formation of Zn-dependent necrosis in transgenic leaves.

## Methods

### Plant material and growth conditions

The experiments were performed on wild-type tomato (*Lycopersicon esculentum* L. var. Beta) and two homozygous lines (nos. 4 and 15) of transgenic tomato expressing 35S::*AtHMA4* from *Arabidopsis thaliana* with similar expression level of the transgene*.* These two lines were chosen as they differ in the extent that Zn root/shoot distribution, which is higher in line 4 [13]. The seeds of the wild-type tomato were obtained from the stock of the Warsaw University of Life Sciences, and used as the parental line for transformation [13].

Seeds were surface-sterilized in 70 % ethanol and rinsed with sterile deionized water. Germination and the first stage of seedling development took place for 10 days in glass jars containing the basic medium supplemented with 2 % (*w/v*) sucrose solidified with 1 % agar (*w/v*). The obtained 10-day-old seedlings were transferred to 2-L pots (six seedlings per pot) for further growth under hydroponic conditions. They were cultivated on quarter-strength Knop’s medium supplemented with different concentrations of Zn (as ZnSO_4_) and Cd (as CdCl_2_), depending on the experiment (detailed information in the subsections below). The nutrient solution was replaced every two days. Quarter-strength Knop’s medium (containing 0.5 μM Zn) was used as the reference control medium. Plants were grown in a growth chamber under the conditions described by Kendziorek [13].

### Cryosectioning, laser microdissection, and microarray

Transgenic line 4 displaying the strongest reduction of the Zn level in the roots and enhancement in the shoots [13] was used in the experiment. Ten-day-old seedlings were grown on liquid quarter-strength Knop’s medium containing 5 μM Zn for one week and root and leaf fragments were collected for laser microdissection, RNA isolation, and microarray analysis. Three independent experiments were performed. Each experiment was performed on six plants, collecting ten roots and two leaves per plant. Collected root and leaf samples were frozen, sectioned in a cryostat, followed by Laser Capture Microdissection (LCM). Two sectors were isolated from roots: epidermis + cortex (EC), and stele (S). The palisade parenchyma + upper epidermis (EPP) and spongy parenchyma + lower epidermis (ESP) were obtained from leaves. These tissue samples were used for RNA isolation and amplification. The aRNA (RNA amplified using RiboAmp HS Kit, details in Additional file 1) was used for the microarray transcriptome experiment and analysis. GeneChip Tomato Genome Array (Affymetrix) microarrays consisting of over 10 000 *L. esculentum* probe sets were used (details in Additional file 1).

In order to confirm the validity of the microchip data, quantitative real-time PCR (RT-qPCR) analysis of the expression of chosen genes was performed. Plants grown under the same conditions as those in the microarray experiment were used for this purpose.

Moreover, roots and leaves were collected for determination of Zn concentration, to confirm the difference in the Zn root/shoot distribution between the wild-type and transgenic line no.4 detected by Kendziorek [13].

### Determination of Zn localization in leaves

Zn in leaves of transgenic (line 4) and wild-type tomato was localized using Zinpyr-1 as a Zn indicator [15]. Ten-day-old plants grown on agar-solidified medium were transferred to liquid quarter-strength Knop’s medium containing 0.5 and 5 μM Zn for one week. The 3^rd^ and 4^th^ leaves were collected, and transverse cross-sections through the whole leaf were made (~1.5 mm thick) at a distance of 2 cm from the base of the blade. Sections were exposed to 10 μM  Zinpyr-1 in NaCl 0.9 % for 1.5 h in the dark. Technical details are given in Siemianowski [15]. The fluorescent signal of Zinpyr-1-bound Zn was monitored under a Nikon A1 confocal laser scanning microscope (Melville, NY, USA) [excitation at 488 nm, emission between 500 and 550 nm]. Each cross section was analyzed by the examination of consecutive sectors to gain the entire picture of Zn localization.

### Comparative study of metal accumulation and accompanying tissue-specific gene expression

Plants were exposed to combinations of Zn and Cd concentrations. Ten-day-old plants were transferred from agar-solidified medium for 14-day exposure to liquid quarter-strength Knop’s basic medium containing the following combinations of Zn and Cd concentrations: (i) 0.5 μM Zn (basic medium); (ii) 5 μM Zn; (iii) basic medium (3 days) followed by 11-day exposure to 1 μM Cd added to the basic medium. The root fragments were collected for laser microdissection, RNA isolation, and RT-qPCR expression analysis, and for determination of Zn and Cd concentrations. Three experimental trials were performed; in each, ten roots were collected from six plants.

Root apical fragments  1.5 cm in length were excised and then 2-cm long fragments were fixed in pre-chilled methanol:acetic acid (3:1 v:v) overnight (approx. 16 hours) at −20 °C (modified method described by Kozubek [16]) and embedded in Steedman wax [17]. Sectioning was performed at −5 °C in an HM 560 cryostat (Microm). The ArcturusXT™ LCM System (Arcturus Engineering, CA) was used to isolate epidermis + cortex (EC) and stele (S) from root sections. The samples were stored at −80 °C for further RT-qPCR analysis. Methodological details are presented in Additional file 2.

### Real-time RT-qPCR

Quantitative real-time reverse transcription–PCR (RT–qPCR) was conducted on aRNA (details on isolation and amplification of RNA in Additional file 1). RNA isolated from LCM-derived tissues of high quality was used for experiments (Additional file 3). The cDNA used as a template for the RT-qPCR reaction was synthesized using RevertAid™ First Strand cDNA Synthesis Kits (Fermentas) in a 20 μl reaction volume containing 0.1–1 μg of aRNA and oligo d(T)18 primers following the manufacturer’s protocol. RT–qPCR was performed in a MyiQ™ 2 cycler (Bio-Rad Laboratories Inc. Hercules, California, USA) using Platinum SYBR Green qPCR superMix-UDG (Invitrogen) according to the manufacturer’s protocol. The reaction was carried out in a volume of 15 μl containing 40 nM primers.

The specific primers (Additional file 4) used in the reactions were designed using IDT OligoAnalyzer 3.1 (http://eu.idtdna.com/calc/analyzer) and OligoCalc:Oligonucleotide Properties Calculator (http://www.basic.northwestern.edu/biotools/oligocalc.html). To test the efficiency of primers used in RT-qPCR, the reaction was carried out for four different concentrations of cDNA template (dilution factor: 10, 10^2^, 10^3^ and 10^4^) obtained on the basis of total RNA isolated from tomato roots or leaves. Standard curves were obtained and reaction efficiency calculated using BioRad iQ4 software.

Expression analysis was performed with at least three independent biological replicates. For each sample, reactions were set up in triplicate and means were calculated. The tomato *LeCyP* (cyclophilin) gene was used as the reference gene/internal control and was amplified in parallel with the target gene allowing gene expression normalization and providing quantification. The stability of the reference gene under the applied conditions was determined (Additional file 5). Quantification of the relative transcript levels was performed using the comparative Ct (threshold cycle) method.

### Determination of elemental concentrations

Zn and Cd concentrations were determined in roots and leaves collected at the end of each experiment. Roots were washed briefly with deionized water, next with 5 mM CaCl_2_ (10 min) at 4 °C to remove unbound and weakly bound metal from the apoplast, then again twice with water (5 min each). Petioles were removed from the leaves and leaflets were collected. Plant samples were oven dried for 4 days at 55 °C, homogenized, then acid digested in a mixture of 65 % HNO_3_ and 39 % H_2_O_2_ (9:1, *v/v*) in a closed system microwave mineralizer (Milestone Ethos 900, Milestone, Bergamo, Italy). Measurements were done by atomic absorption spectrophotometry (TJA Solutions Solar M, Thermo Electron Manufacturer Ltd., Cambridge, Great Britain) according to Barabasz [11].

### Apoplastic fluid analysis

Ten-day-old transgenic (lines no. 4 and 15) and wild-type plants grown on agar-solidified medium were transferred to hydroponic conditions for 12 days, then exposed to 5 μM Zn for 2 days and used for the determination of Zn and K concentrations in the apoplastic fluid. Two-day exposure to 5 μM Zn was chosen because longer one causes tissue stiffening and decreases the efficiency and quality of apoplastic fluid isolation. The 3^rd^ and 4^th^ leaves were collected from each plant (5 plants per trial, three repetitions). Apoplastic fluid was collected and elemental concentrations determined according to Barabasz [11].

### Statistical analysis

All presented data are from one experiment that is representative of three to four independent replicate experiments. Statistical significance was evaluated at the 0.05 probability level using Student’s t-test (except microarray analysis – details are given in the Additional file 1).

## Results

### Modifications of Zn, Cd, and Fe accumulation in *AtHMA4*-expressing tomato

The homeostasis of Zn, Cd and Fe is not specific for each metal, but is interconnected by common pathways and is called “metal cross-homeostasis”. In transgenic plants ectopically expressing Zn transporters, this phenomenon contributes to alteration of not only Zn accumulation and distribution, but also that of other metals like Cd or Fe [14]. Therefore, although AtHMA4 (a Zn-transporter) was expressed in tomato to improve the Zn content in shoots, the concentration of Cd and Fe in transgenic and wild-type plants was also examined.

**Fig. 1:**
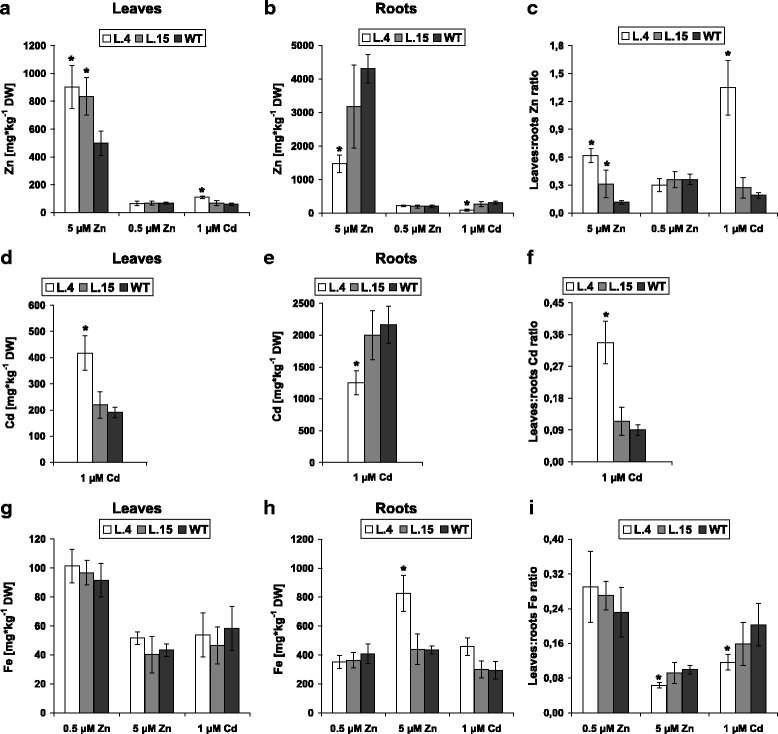
Zn, Cd and Fe concentration in 24-day-old transgenic (lines 4 and 15) and wild-type plants. Plants were exposed to 5 and 0.5 μM Zn (control) for 2 weeks and to 1 μM Cd for 11 days. Zn concentration in leaves (**a**); roots (**b**); leaves/roots Zn concentration ratio (**c**). Cd concentration in leaves (**d**); roots (**e**); leaves/roots Zn concentration ratio (**f**) of Cd-exposed plants. Fe concentration in leaves (**g**); roots (**h**); leaves/roots Fe concentration ratio (**i**). Values correspond to means ± SD (*n* = 6); those significantly different from the WT (Student’s t-test) are indicated by an asterisk (*P* ≤ 0.05)

Expression of *AtHMA4* facilitates root-to-shoot Zn translocation but in a Zn supply-dependent manner. In plants grown at 5 μM Zn, the expression of *AtHMA4* enhanced the translocation of Zn to shoots and its concentration in leaves, but the differences versus wild-type plants were much more pronounced in line 4. However, upon exposure to 0.5 μM Zn, there was no difference in Zn accumulation among the studied lines (Fig. 1a-c).

Interestingly, when Cd was added to the control medium (containing 0.5 μM Zn), Zn translocation to leaves of transgenic plants was stimulated, especially in line 4 (Fig. 1c). Expression of *AtHMA4* significantly increased the Cd concentration in leaves and reduced it in roots, but only in line 4 (Fig. 1d-f). The total Fe concentrations did not differ much between transgenic and wild-type plants, both in the leaves and in the roots for most examined experimental variants (Fig. 1g-i).

In summary, the level of Zn in the shoots of tomato expressing *AtHMA4* (responsible for the control of Zn translocation to shoots in *A. thaliana*) depended on Zn supply, and also on the presence of Cd. This points to the difficulties in engineering a plant displaying the desired Zn level in shoots (e.g. for biofortification or phytoremediation purposes) when grown under differing conditions of metal supply. To better plan future modifications of metal uptake and root/shoot distribution, it is necessary to unravel the molecular mechanisms induced in transgenic plants that are responsible for the detected diverse phenotype of transgenics grown on media of varying mineral compositions. Therefore, detailed expression analysis of transgenic and wild-type plants was performed.

### Roots

#### Microarray-based analysis of the transcription profiles of LCM-derived tissues from roots of transgenic and wild-type tomato exposed to 5 μM Zn

As a first step, microarray analysis was used to identify the endogenous tissue-specific molecular metal-cross-homeostasis pathways that are activated in the roots of tomato expressing *AtHMA4* and which accompany increased Zn root-to-shoot translocation. The transcription profiles of root tissues collected by LCM from transgenic line 4 (with Zn translocation to shoots enhanced more efficiently than in line 15) and wild-type plants grown at 5 μM Zn were compared (Fig. 1a-c). The Zn concentration in the root tissues used for expression analysis is shown in Additional file 6.

Transcript profiles were compared in two root sectors: epidermis + cortex (EC) and the stele (S). Gene expression was considered as up- or downregulated if the transcript level showed a minimum of a 1.2-fold change and FDR of <0.05 (a low value was chosen to avoid overlooking smaller changes which might also be important in modifications of Zn root/shoot distribution in *AtHMA4*-expressing plants; RT-qPCR was used as final confirmation of a difference) (Additional file 1). The complete dataset is available in the NCBI with accession no. GSE68641 (http://www.ncbi.nlm.nih.gov/projects/geo/query/acc.cgi?acc=GSE68641), and is also included as Additional file 7). The NCBI Accession Numbers of nucleic acid sequences are given in the appropriate Additional files.

**Fig. 2:**
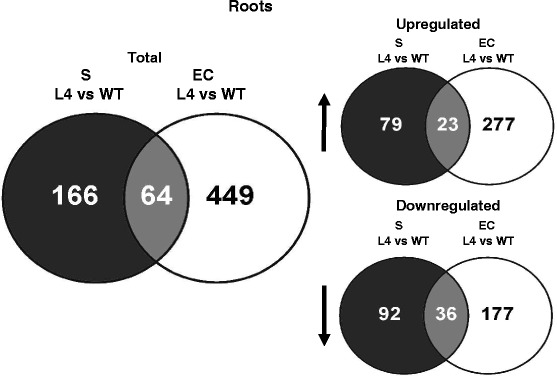
Venn diagrams illustrating significant transcriptional changes in the stele (S) and in the epidermis+cortex (EC) from roots of AtHMA4 transformants relative to wild-type (WT)

**Fig. 3:**
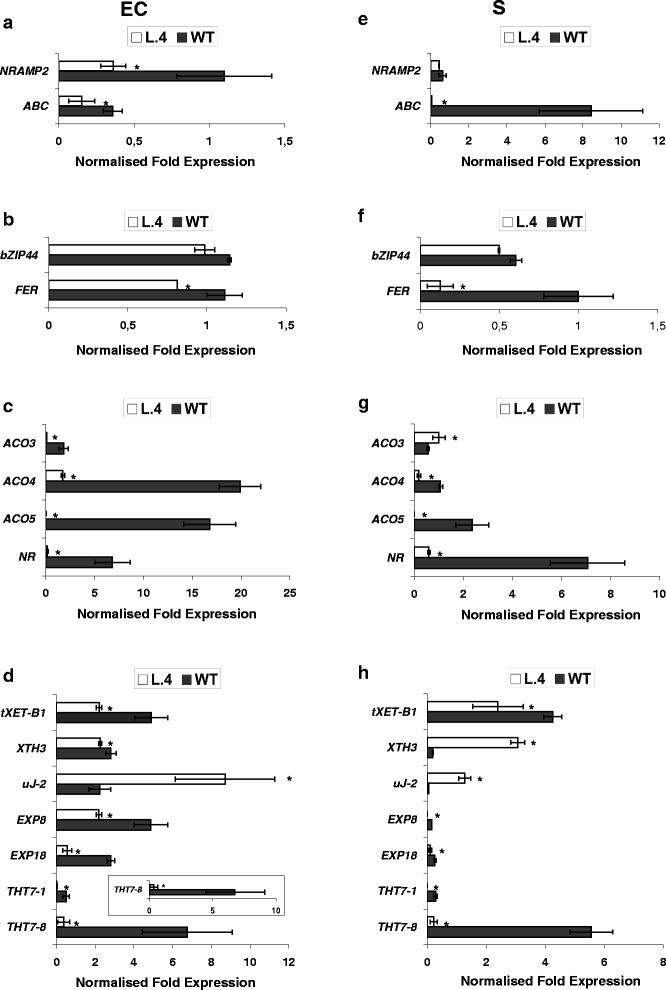
Confirmation of microarray results by quantitative real-time PCR. The chosen genes identified by microarray analysis as differentially expressed in the epidermis + cortex (EC) and stele (S) from roots of 17-day-old AtHMA4-expressing plants (lines 4) and in the wild-type (WT) grown in the presence of 5 μM Zn for one week. Normalized fold gene expression for (**a**) metal transporters and uptake facilitators; (**b**) transcription factors; (**c**) cell wall modification; (**d**) ethylene pathway genes. Values correspond to means ± SD (*n* = 3); those significantly different from the WT (Student’s t-test) are indicated by an asterisk (*P* ≤ 0.05)

In *AtHMA4*-expressing tomato, as many as 513 genes were differentially expressed in the EC, whereas a lower number, 230 genes, was detected in the S (Fig. 2). The relative distribution of differentially expressed genes in roots classified by GO (Gene Ontology) of biological processes (using the Tomato Functional Genomics database) (Additional file 8) shows that genes related to categories such as “metabolic processes”, “cellular processes”, “response to stress”, and “transport” constitute a relatively large group, indicating importance in the regulation of a transgenic plant’s response to the applied conditions. Our focus was on differentially expressed genes involved in: (i) metal transport; (ii) transcription factors; (iii) ethylene biosynthesis; (iv) cell wall modifications. The complete sets of genes for these four categories present in the microarray are shown in Additional file 9. The genes selected for further analysis are listed in Additional file 10, and the microarray data were validated by RT-qPCR (Fig. 3) confirming differential expression for most of the selected genes (Additional file 10 and (Fig. 3). Some inconsistencies between the microarray and RT-qPCR expression analyses were most likely due to the lower specificity of the microarray probes than of the gene-specific primers used in RT-qPCR. The expression of certain genes selected for detailed analysis was found by microarray to be higher, and by RT-qPCR to be lower than in the wild type. The probes for these genes also recognize other sequences: (i) *LeNRAMP2*: probe Les.4532.1.S1_at recognizes fragments of chromosomes 05, 07, 10, 12; (ii) *LeFER*: probe Les.3814.1.S1_at recognizes fragments of chromosomes 02, 05, 07; (iii) *LebZIP44*: probe Les.3126.1.S1_at also recognizes fragments of chromosome 05. Thus, if the outcome of the RT-qPCR-based expression study differed from that of the microarray experiment, the results of the more sensitive and reliable RT-qPCR analysis were accepted as valid.

Two metal transporters were identified (Fig. 3a, e). The first was *LeNRAMP2* (Natural Resistance-Associated Macrophage Protein), with a slightly lower level of mRNA in the EC of transformants. The second was ABC putative transporter (ATP-Binding Cassette transporter) strongly downregulated in the S of transgenic plants relative to the wild type.

In the category of transcription factors, *LeFER* (encoding the bHLH protein) was identified as a gene whose mRNA levels in transgenic plants were lower in both root sectors than in the wildtype*.* On the other hand, *LebZIP44* (basic-region leucine-zipper) mRNA remained at the wild-type level (Fig. 3b, f).

Interestingly, profound downregulation of most identified biosynthesis genes and ethylene receptors was found in transgenic plants relative to the wild type (Fig. 3c, g). Ethylene receptor *NR (never-ripe)*, *LeACO4*, and *LeACO5* (encoding 1-aminocyclopropane-1-carboxylate oxidases) were strongly downregulated in the EC and in the S, but *LeACO3*, in the EC only.

Moreover, in transgenic vs. wild-type plants, microarray data indicated differential expression of numerous genes involved in cell wall remodeling (Additional file 10). Considering the importance of the apoplast in a plant’s response to metals [18, 19], we focused on seven genes with the most pronounced expression modifications. RT-qPCR analysis (Fig. 3d, h) showed downregulation of *LeTHT7-1*, *LeTHT7-8* (N-hydroxycinnamoyl-CoA:tyramine N-hydroxycinnamoyl transferase)*, LeExp8, LeExp18* (expansins), and *LetXET-B1* (xyloglucan endotransglucosylase), and upregulation of *LeuJ-2* (an extensin) in both examined tissues. For *LeXTH3* (xyloglucan endotransglucosylase/hydroxylase) the transcript level was higher in the S of transformants than in the wild type.

#### RT-qPCR-based analysis of gene expression in LCM-derived tissues from roots of transgenic and wild-type plants exposed to different Zn concentrations and to Cd

**Fig. 4:**
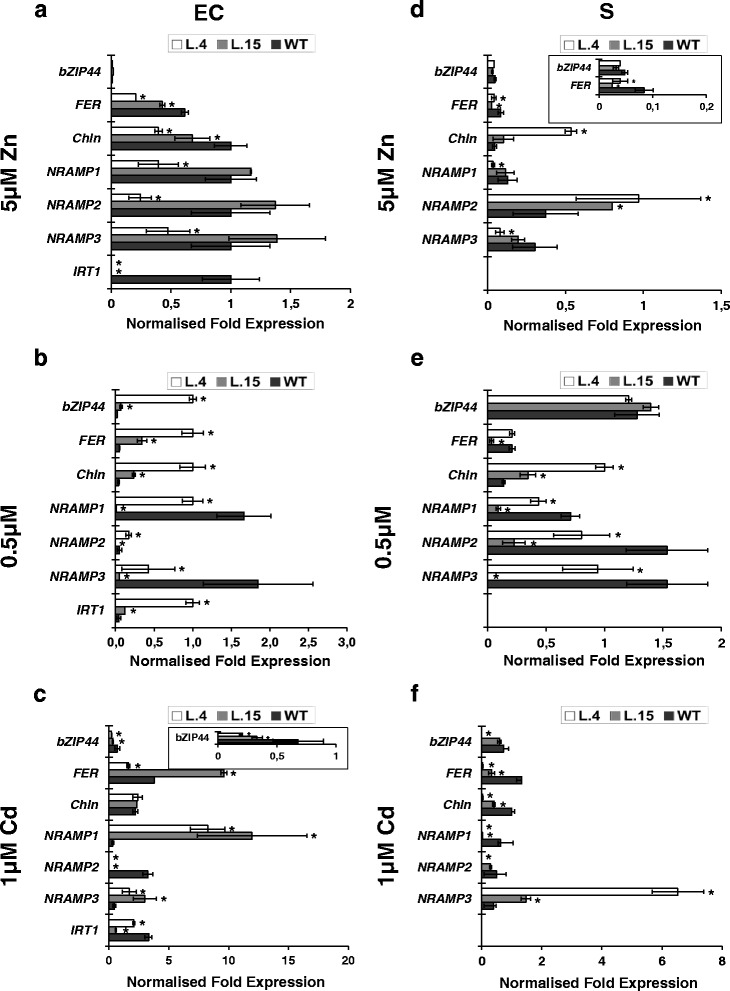
Tissue-specific transcription profiles of chosen genes involved in metal homeostasis differentially expressed in epidermis + cortex (EC) and stele (S) of roots of 24-day-old *AtHMA4*-expressing plants (lines 4 and 15) and in the wild-type (WT). Normalized fold gene expression in epidermis + cortex (EC) and stele (S) from roots of plants grown in the presence of 5 μM Zn for 2 weeks (**a**; **d**); under control conditions (**b**; **e**) and in the presence of 1 μM Cd for 11 days (**c**; **f**). In the EC from roots of transgenic line 15, the transcription levels of the studied genes were hardly detectable (values were as follows: *IRT1* = 0; *NRAMP1* = 0; *NRAMP2* = 0; *NRAMP3* = 0; *Chln* = 0.005873; *FER* = 0.0067678; *bZIP44* = 0). *LeIRT1* is not expressed in the S. Values correspond to means ± SD (*n* = 3); those significantly different from the WT (Student’s t-test) are indicated by an asterisk (*P* ≤ 0.05)

At this stage of the research, the aim was to identify the molecular mechanisms behind the Zn- and Cd-supply-dependent modifications of Zn and Cd accumulation patterns in transgenic plants (Fig. 1). We examined the possible contribution of metal-homeostasis and ethylene-related genes (except cell wall-remodeling genes) (Fig. 3) that were selected from among the genes identified in the microarray-based analysis. In addition, four metal-homeostasis genes were included in the expression study (Additional file 11): (i) *LeIRT1* (Iron Regulated Transporter 1, dependent on *FER*), the Strategy I Fe-deficiency uptake gene [20] known to mediate uptake of Zn and Cd; (ii) *LeNRAMP1* and *LeNRAMP3* involved in metal redistribution [20]; (iii) *LeChln* (*LeNAS*), encoding nicotianamine synthase participating in the biosynthesis of nicotianamine (NA), a metal chelator involved in the regulation of Zn and Cd translocation to shoots [20, 21]. In this study two transgenic lines were compared—lines 4 and 15. They differ in the efficiency of Zn translocation to shoots (higher in line 4) (Fig. 1) [13]. The expression pattern of the examined endogenous genes was substantially modified in transgenic plants as compared with the wild type (Fig. 4).

There was also a major difference in the expression pattern between transgenic plants grown at 5 and 0.5 μM Zn. In the EC, the expression of ethylene-related genes *LeACO4, LeACO5*, the ethylene receptor *LeNR*, as well as the ethylene-dependent *LeChln* and the Fe (also Zn and Cd) acquisition system represented by *LeFER*, *LeIRT1* was lower (relative to the wild type) in plants exposed to 5 μM Zn, but higher in those grown at 0.5 μM Zn (Fig. 4a, b). The expression of *NRAMPs* in the EC was lower for both tested Zn conditions, but primarily for line 4, except *LeNRAMP2*.

In the S, the transcript level in transgenics grown at both 0.5 and 5 μM Zn was lower than in the wild type for most of the tested genes, except differential expression of *LeNRAMP2* (Fig. 4d, e).

**Fig. 5:**
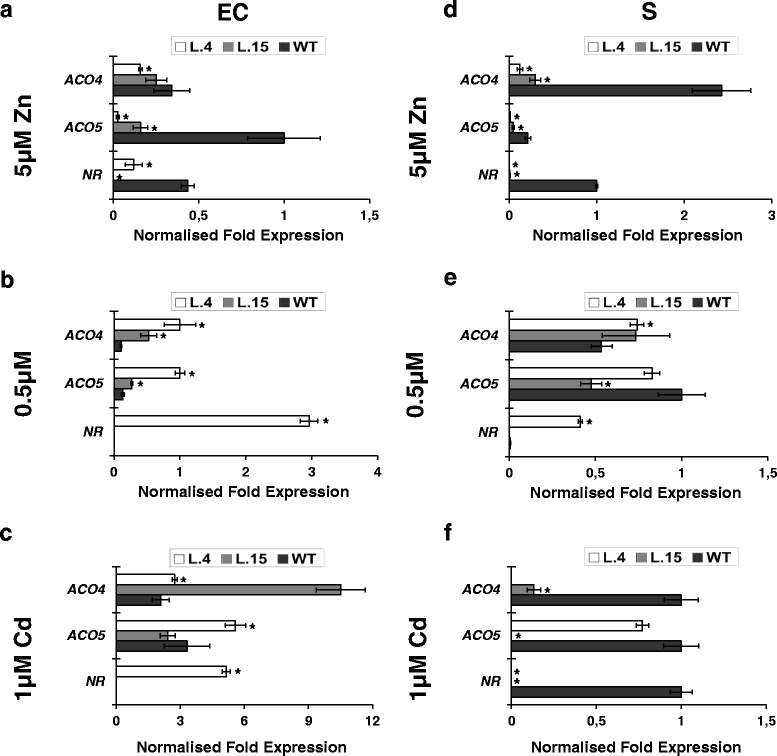
Tissue-specific transcription profile of genes involved in the ethylene pathway differentially expressed in epidermis + cortex (EC) and stele (S) of roots of 24-day-old *AtHMA4*-expressing plants (lines 4 and 15) and in the wild-type (WT). Normalized fold gene expression in epidermis + cortex (EC) and stele (S) from roots of plants grown in the presence of 5 μM Zn for 2 weeks (**a**; **d**); under control conditions (**b**; **e**) and in the presence of 1 μM Cd for 11 days (**c**; **f**). Values correspond to means ± SD (*n* = 3); those significantly different from the WT (Student’s t-test) are indicated by an asterisk (*P* ≤ 0.05)

When 0.25 μM Cd was added to the control medium containing 0.5 μM Zn, in the EC the expression pattern of *LeNR*, *LeACO4*, and *LeACO5* remained similar to controls for all tested lines; the opposite was observed, however, for *NRAMP*s. In the S of transgenic plants lower expression than in the wild type was detected both for ethylene genes (*LeNR*, *LeACO4*, and *LeACO5*) and for ethylene-dependent *LeFER, LeChln*, and *LeNRAMP1* (Fig. 4c, f; Fig. 5c, f).

### Leaves

#### Microarray-based analysis of the transcription profiles of LCM-derived tissues from leaves of transgenic and wild-type tomato exposed to 5 μM Zn

**Fig. 6:**
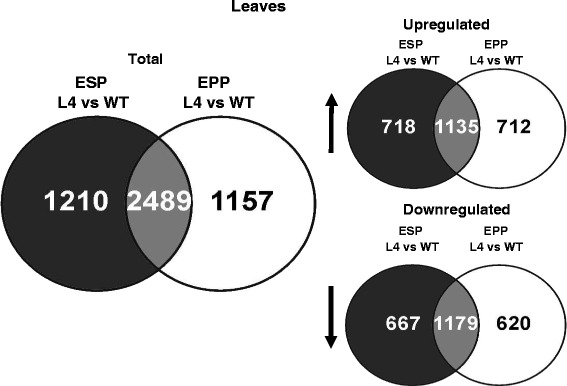
Venn diagrams illustrating significant transcriptional changes in spongy parenchyma + lower epidermis (ESP) and palisade parenchyma + upper epidermis (EPP) from leaves of *AtHMA4* transformants relative to wild-type (WT)

**Fig. 7:**
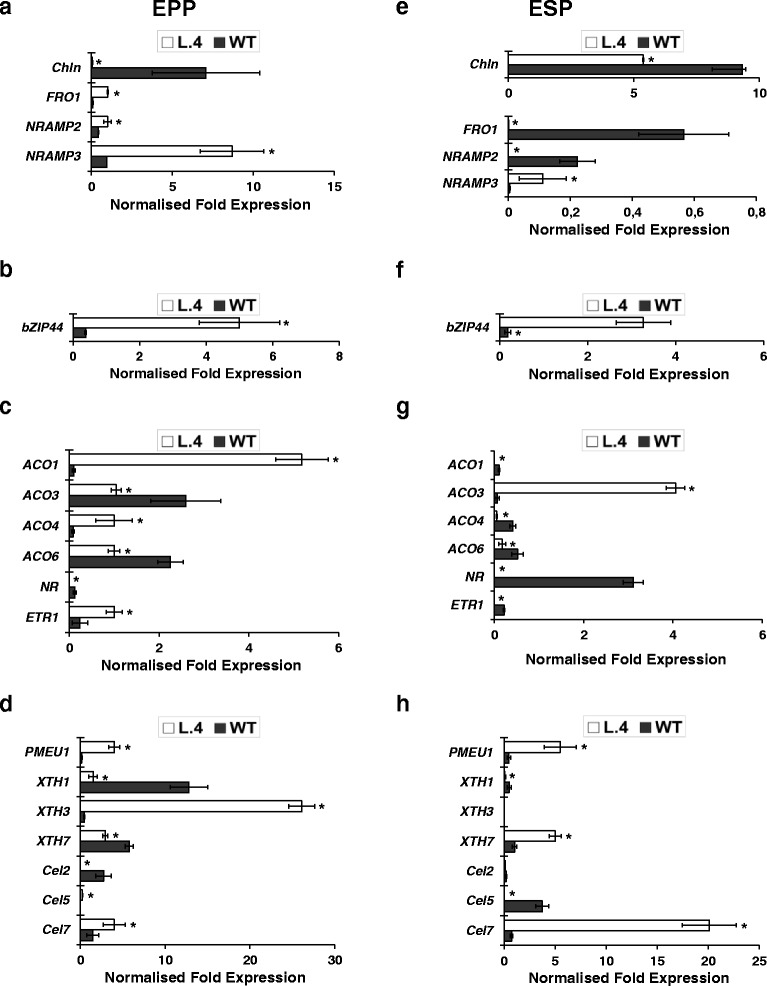
Confirmation of microarray results by quantitative real-time PCR. The chosen genes identified by microarray analysis as differentially expressed in palisade parenchyma + upper epidermis (EPP) and spongy parenchyma + lower epidermis (ESP) from leaves of 17-day-old *AtHMA4*-expressing plants (line 4) and in the wild-type (WT) grown in the presence of 5 μM Zn for one week. Additionally, the expression of *LeChln* was analyzed. Normalized fold gene expression for **a**, **e** metal transporters and uptake facilitators as well as nicotianamine biosynthesis genes; **b**, **f** transcription factors; **c**, **g** cell wall modification; **d**, **h** ethylene pathway genes. Values correspond to means ± SD (*n* = 3); those significantly different from the WT (Student’s t-test) are indicated by an asterisk (*P* ≤ 0.05)

It was found that in the ESP and the EPP of transgenic plants exposed to 5 μM Zn, the transcription levels of 3699 and 3646 genes, respectively, were altered relative to the wild type (Fig. 6). Among GO categories, the most prominent represented “cellular processes”, “transport”, and “responses to stresses” (Additional file 12), indicating activated endogenous pathways due to *AtHMA4* expression. The same gene categories as in roots were chosen for further analysis, and RT-qPCR confirmed differential expression of the majority of them. Similarly as in roots, in cases when the RT-qPCR analysis did not confirm the expression pattern indicated by microarray, the results of the RT-qPCR analysis were accepted as valid (Additional file 13; Fig. 7). The most significant changes in the tissue-specific expression of the examined genes due to expression of *AtHMA4* include a marked increase in the mRNA level of *LeNRAMP3* in the EPP and a decrease of *LeChln*. Moreover, strong upregulation of *bZIP44* was detected in both leaf sectors (Fig. 7a-b, e-f).

Substantial changes in the expression of genes from the ethylene pathway were also noted (Fig. 7c, g). The mRNA of two ethylene receptors had lower levels than the wild type in both analyzed tissues (*LeNR*) or in the ESP only (*LeETR1).* It is noteworthy that their expression was significantly different in the wild type, where it was low for *LeETR1* in both tissues and high for *LeNR* in the ESP. On the other hand, the transcript abundance of four identified *ACOs* in transgenic plants differed in the studied leaf sectors. The most dramatic increases were shown for *LeACO1* and *LeACO4* in the EPP, and for *LeACO3* in the ESP. Moreover, in transgenic plants, significantly lower transcript levels than in the wild type in both tissues were shown for *LeACO6*.

The expression of *AtHMA4* also resulted in marked changes in the expression of genes involved in cell wall modifications (Fig. 7d, h). First, strong upregulation of *PMEU1* (pectin-methyl-esterase) in both tissues was noted. The second class was *XTH*. Three of these genes (*XTH1, XTH3, XTH7*) were differentially expressed in the transgenic plants, however, not in the same fashion. The third class was the endo-1,4-beta-D-glucanases (*Cel* genes). In both analyzed tissues, lower and higher transcript levels were found in transgenic plants for *LeCel2* and *LeCel7,* respectively.

Thus, ectopic expression of *AtHMA4* in tomato modified transcription profiles of the studied genes in a tissue-specific manner with striking differences between the EPP and the ESP in certain cases.

#### AtHMA4 expression overloads the apoplast of transgenics’ leaves with Zn and modifies Zn distribution in the leaf blade

**Fig. 8:**
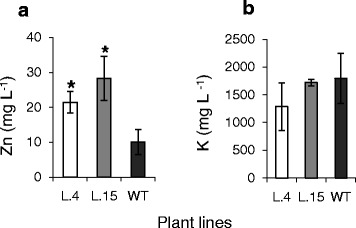
Zn and K concentrations in leaf apoplastic fluid. Apoplastic fluid was isolated from 24-day-old transgenic (lines 4 and 15) and wild-type (WT) plants grown for 2 days in the presence of 5 μM Zn. Zn concentrations (**a**); K concentrations (**b**). Values correspond to means ± SD (*n* = 5); those significantly different from the WT (Student’s t-test) are indicated by an asterisk (*P* ≤ 0.05)

It is known that AtHMA4 exports Zn to the apoplast [2,3,10]. Here we confirmed that expression of *AtHMA4* in tomato enhanced the Zn concentration in the apoplast of leaves (Fig. 8a). Thus, the Zn status at the cellular level in transgenic plants was different from that of the wild type. The potassium concentration, used as an indicator of cytoplasmic contamination, remained at comparable levels in all tested lines (Fig. 8b).

In our previous study on *AtHMA4*-expressing tobacco we showed that the enhanced concentration of Zn in the apoplast of leaves from plants grown in the medium containing a high concentration of this metal is related to the initiation of Zn loading into clusters of “Zn storage” mesophyll cells [19]. Therefore, in this study, finding that expression of *AtHMA4* in tomato modifies the expression of endogenous genes and changes the Zn apoplast/symplast status, prompted us to examine the Zn distribution pattern within leaves.

**Fig. 9:**
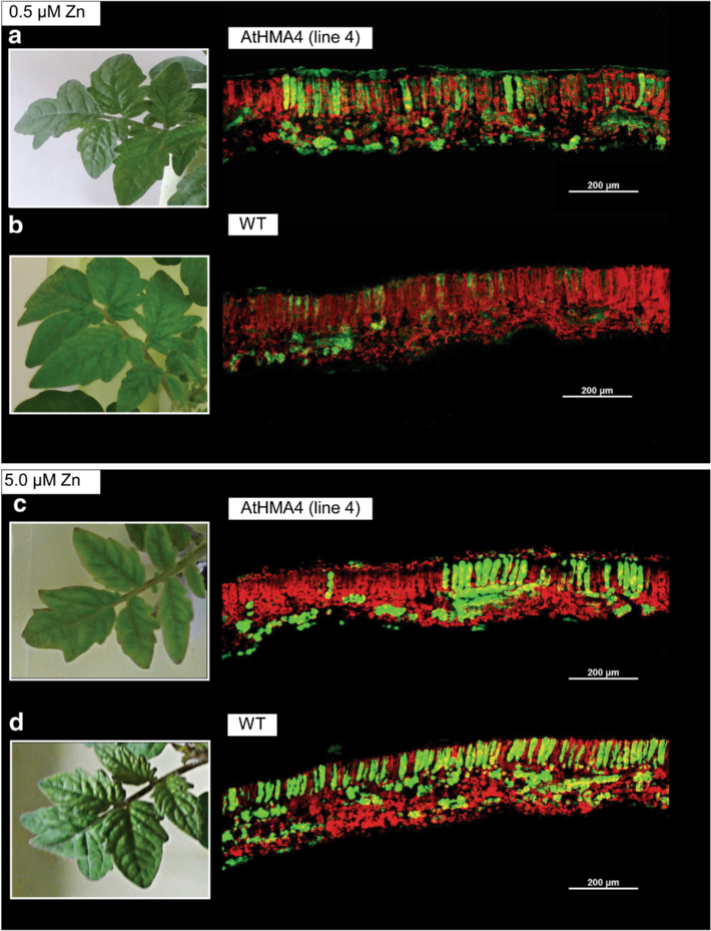
Zn distribution at cross sections through leaves visualized by Zinpyr-1. Cross sections made through leaves of 17-day-old wild-type (**a**, **c**) and *AtHMA4*-expressing tomato plants from line 4 (**b**, **d**) grown for one week in the presence of 0.5 μM Zn – control conditions (**a**, **b**), and 5 μM Zn (**c**, **d**). Confocal laser scanning microscope (CLSM) settings were different for sections made from leaves grown under control conditions and followed by 5 μM Zn due to the stronger fluorescence of leaves exposed to high Zn. Magnification bars are indicated in the figure

The presence of Zn was indicated by Zinpyr-1-based green fluorescence (Fig. 9). In the leaves of wild-type plants grown either at 0.5 or 5 μM Zn in the medium, Zn was evenly distributed in the mesophyll cells, though its level was higher at 5 μM Zn (Fig. 9b, d).

In contrast, in transgenic leaves, enhanced Zn levels were detected inside groups of cells (Fig. 9a, c). This is especially evident in plants exposed to 5 μM Zn. Zinpyr-1-based intensive fluorescence indicating a high Zn level was detected in groups of mesophyll cells (primarily palisade parenchyma), whereas neighboring groups of cells showed fluorescence of lower intensity (indicating lower Zn concentrations) (Fig. 9c). Thus, similarly as in tobacco [19], expression of *AtHMA4* in tomato contributed to the manifestation of heterogeneity in the mesophyll cells with respect to their capacity for Zn storage.

## Discussion

### Zn supply-dependent modification of Zn root/shoot distribution due to expression of *AtHMA4* in tomato is accompanied in roots by tissue-specific differential expression of metal-homeostasis genes

**Fig. 10:**
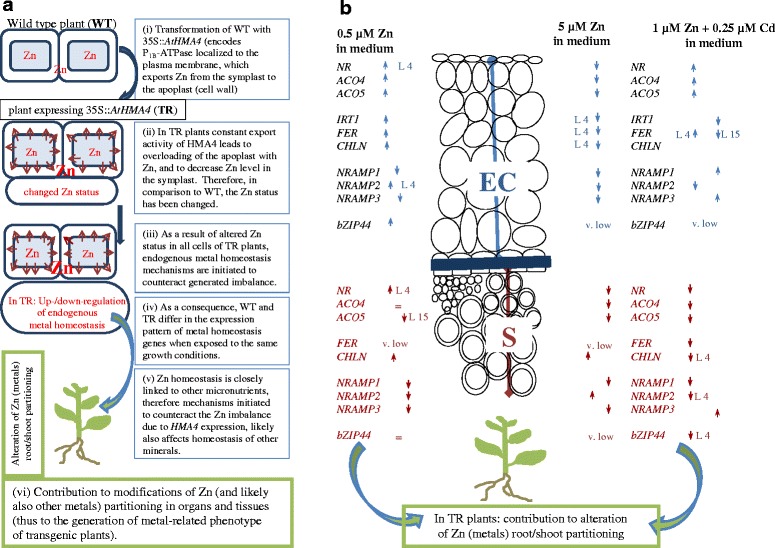
Model explaining changes in the expression pattern of endogenous metal homeostasis genes in *AtHMA4*-expressing plants, that contribute to the generation of the phenotype of transformants. **a** Effect of ectopic expression of 35S::*AtHMA4* on cellular Zn status of the host plant, followed by modification of the expression pattern of endogenous homeostasis genes - starting point for the current research. **b** Molecular consequences of altered Zn status due to constant export activity of AtHMA4 in transgenic tomato (TR) exposed to 0.5 μM Zn, 5 μM Zn and to 0.5 μM Zn + 0.25 μM Cd: changes in the expression pattern of endogenous metal-homeostasis and ethylene genes in TR vs wild-type (WT) are Zn-supply dependent, Cd-dependent and tissue-specific; Exposure of WT to lower/higher Zn in the medium generates a Zn-status-specific transcriptome background; In TR plants, a combination of a given Zn-status-specific generated molecular background (resulting from the Zn level in the medium) with changes in the Zn status resulting from the constant export activity of AtHMA4, contributes to the detected differences in the tissue-specific expression pattern of the examined genes. The presence of Cd changes also the tissue-specific expression pattern in TR in a fashion different from that of WT. EC- epidermis + cortex; S – stele

Our previous study showed that expression of *35S::AtHMA4* and *AhHMA4*_*p*_*::AhHMA4* in tobacco and tomato led to changes in Zn and Cd accumulation and root/shoot distribution. The pattern was different, however, at a range of metal concentrations in the medium, indicating involvement of endogenous processes specifically induced in transgenic plants [10–13]. Here it was shown that the Zn supply-dependent modifications of Zn accumulation due to *AtHMA4* expression (increased Zn root-to-shoot translocation at 5 μM Zn, no change at 0.5 μM Zn) were accompanied in the roots by tissue-specific expression patterns of metal-homeostasis genes that were different than in the wild type. Importantly, the pattern of modifications detected in transgenic plants (as compared with the wild type) was not the same upon exposure to 0.5 and 5 μM Zn in the medium. According to the presented model (Fig. 10), changes in the expression of endogenous genes in transgenic plants are induced in response to the mineral imbalance resulting from the export activity of AtHMA4 in transgenic tomato. These alterations contribute to the generation of the phenotype of transgenic plants, including changes in the accumulation and distribution of metal(s) between roots and shoots. It has already been shown [22] that plants grown under different conditions of exposure to metals (metal deficiency, sufficiency, and excess) differ greatly in their expression profiles. Therefore, it was assumed that these very different molecular backgrounds of tomato (used for transformation) grown at varying Zn levels in the medium will interact with the changes in the mineral status at the cellular level (resulting from AtHMA4 export activity), the extent of which is different at lower and higher Zn exposure. As a result, in specific cells/tissues/organs endogenous genes known to respond to the availability of a metal(s) are up- or downregulated in transgenic plants in a tissue-dependent fashion. The activation of endogenous metal cross-homeostasis mechanisms in transgenic plants is considered a key factor that contributes to the generation of their characteristics.

#### Changes specific for the EC (epidermis + cortex)

In numerous plant species, Zn excess decreases the Fe level in aerial parts and induces an Fe-deficiency response due to a shoot-born systemic signal [22–24]. In the wild-type tomato, the shoot Fe content was lower at elevated Zn (10 and 20 μM) compared with 1 μM Zn [13]. In line with these observations, here it was shown that in the EC of roots from wild-type tomato grown at 5 μM Zn, the expression of transcription factor *LeFER,* and *LeFER*-dependent *LeIRT1* that mediates Fe, Zn and Cd uptake [20, 25, 26] was higher compared with 0.5 μM Zn exposure (Fig. 4a, b; Fig. 5a, b). However, expression of *AtHMA4* changed the intracellular Fe status, which was different at the lower and higher Zn levels in the medium.

Thus, expression of the Fe deficiency-inducible Strategy I Fe acquisition genes (*LeIRT1* and *LeFER*), ethylene genes (*LeNR*, *LeACO4*, and *LeACO5*), and *Chln* found to be higher in transgenic plants than in the wild type grown at 0.5 μM Zn, indicates generation of Fe deficiency status due to *AtHMA4* expression. Conversely, the lower expression of these genes in AtHMA4-plants grown at 5 μM Zn indicated that their Fe status had changed to Fe sufficiency. These contrasting changes in the expression of endogenous genes show how deeply the Zn export activity of HMA4 alters endogenous metal cross-homeostasis mechanisms (Fig. 10). In transgenic plants they did not lead, however, to marked changes in total root and leaf Fe concentrations, except in transgenic line 4 grown at 5 μM Zn (Fig. 1g-i), suggesting that in most experimental variants, there occurred the induction of pathways counteracting mineral imbalance generated by the export activity of AtHMA4 in tomato.

Ethylene is necessary for upregulation of Fe-uptake genes [27]. The examined genes included *LeNR* (an ethylene receptor that responds to environmental stresses), *LeACO4*, and *LeACO5*. Ethylene is the product of the reaction catalyzed by 1-aminocyclopropane-1-carboxylate oxidase (ACO), therefore, the site of *ACO* expression is considered the best indicator of ethylene production [28, 29]. Ethylene affects Fe deficiency-inducible genes, including *IRT1*, by regulating the *FER* transcription factor level [30]. Expression of *LeIRT1* also depends on the expression of *LeChln*, the only *NAS* gene in tomato involved in nicotianamine (NA) synthesis that is known to regulate cross-homeostasis of Fe, Zn, Mn, Cd, and Ni, among others [26, 31, 32]. Upon exposure to 0.5 or 5 μM Zn, in the EC the expression of *LeIRT1* in AtHMA4-tomato was higher or lower than in the wild type, respectively, and was accompanied by respectively higher or lower expression of *LeChln* (Fig. 4a).

Moreover, in the EC of transgenic plants grown at 0.5 μM Zn, the expression of *NRAMP1* and *NRAMP3* was lower than in the wild type in both lines, whereas at 5 μM Zn—only in line 4 (Fig. 4a; Fig. 10). It cannot be excluded that the detected modified expression of *LeNRAMPs* could contribute to regulation of the amount of Zn available for radial transport and root-to-shoot translocation, thus, to the difference in Zn and Fe root and shoot concentrations detected between lines 4 and 15 (Fig. 1b-c, h-i). The NRAMPs consist of a group of membrane importer proteins that exhibit functional divergence and broad substrate specificity, including Fe, Mn, Ni, Cd, Zn, Pb [33]. However, up to now the tomato LeNRAMP1 and LeNRAMP 3 localized to the internal membranes have only been shown to mediate Mn transport [20]. Other metals, including Zn, were not tested, hence it was not determined if Zn is a substrate. Further research aimed at elucidation of LeNRAMP1 and LeNRAMP3 function is needed to understand the contribution of these proteins to Zn homeostasis and partitioning in organs.

#### Changes specific for the S (stele)

In the S (where Zn is loaded into xylem vessels) the Zn supply-dependent contribution of *AtHMA4* expression to Zn root-to-shoot translocation efficiency (increase at 5 μM Zn, no change at 0.5 μM Zn; Fig. 1c) was accompanied primarily by a distinct expression pattern of *LeNRAMP2*. In plants exposed to 0.5 μM Zn, *LeNRAMP2* was downregulated, and upregulated at 5 μM Zn (Fig. 4d, e). Thus, *LeNRAMP2* was found to respond in the S of both transgenic lines in a unique way to the changes in mineral status resulting from AtHMA4 export activity (see model in Fig. 10). This suggests that *LeNRAMP2* could be considered a candidate gene for involvement in the Zn supply-dependent efficiency of Zn translocation to shoots, which is distinct in transgenic vs. wild-type plants (Fig. 1c). To corroborate this supposition, it is necessary to characterize *LeNRAMP2*. Currently only its sequence is known. The highest level of sequence identity was found between LeNRAMP2 and the *Arabidopsis* NRAMPs from the second sub-family, which include AtNRAMP2-5 (Additional file 14). It shares 74.2 % amino acid identity with AtNRAMP2 which does not complement the *fet3fet4* yeast mutation, indicating the lack of ability to transport Fe [21]. LeNRAMP2 also shows high identity with AtNRAMP3 and TcNRAMP3 (72.1 % and 71.5 %, respectively), and with AtNRAMP4 and TcNRAMP4 (69.6 % and 68.2 %, respectively). They encode tonoplast-localized proteins implicated in the release of Fe, Mn, and Cd from vacuoles [21, 34–36]. It cannot be excluded that LeNRAMP2, as an import protein, is localized to the internal membranes and participates in metal redistribution from intracellular stores. Detailed molecular and functional characteristics are needed, however, to conclude about its specific role in the S relating to modifications of Zn supply-dependent alteration of Zn translocation to shoots in the AtHMA4-tomato.

### Cd-dependent modifications of Zn/Cd root/shoot distribution due to expression of AtHMA4 in tomato involves root tissue-specific alteration of *LeNRAMP1-3* and *LeChln*

Expression of *AtHMA4* also contributed to enhanced Cd root-to-shoot translocation. Moreover, in the presence of Cd, the efficiency of Zn translocation was significantly higher, however, the difference was noted primarily in transgenic line 4 (Fig. 1). These changes were accompanied at the molecular level by differences in the abundance of *LeNRAMP1-3* mRNA, especially in the EC (Fig. 4c, f; Fig. 10) indicating that they were regulated in a tissue-specific manner directly or indirectly by Zn/Cd. It is noteworthy that there is a growing amount of data suggesting the involvement of *NRAMP* transporters in mediating Cd uptake and in intracellular distribution in plants [34, 36, 37]. The changes in Cd and Zn accumulation distinct for line 4 were accompanied by the reduction to a barely detectable level of the expression of *LeNRAMP2, LeChln* (*LeNAS*), and yet uncharacterized transcription factors *LebZIP44* in the S of transgenics’ roots. The role of *NAS* in Zn and Cd translocation to shoots has been shown for *AhNAS2* from *A. halleri* [32, 38]. However, the tissue-specific regulation of *LeChln* upon exposure to a range of Zn and to Cd had not been investigated thus far. The expression of *LeFER* and *LeChln* in the S of transgenics’ roots was lower than in the wild type and corresponded with decreased expression of ethylene-related genes (Fig. 10). In the EC this correlation was not as obvious, however. The role for ethylene in a plant’s response to Cd was indicated in experiments showing higher tolerance to Cd in the *Nr* tomato mutant [39], as well as in the *etr1-1* and *ein2-1 Arabidopsis* mutants [40].

### In leaves, ectopic expression of *AtHMA4* modifies the ethylene-dependent pathway in a tissue-dependent fashion

Expression of *HMA4* in tobacco and tomato led to the appearance of necrosis within leaf blades when plants were exposed to elevated Zn in the medium [11,12]. It was shown that loading of Zn into “Zn-storage cells” was initiated upon a high Zn concentration in the apoplast, however, the nature of the signal was not proposed. In this study, in the leaves of AtHMA4-tomato plants grown at moderately toxic 5 μM Zn its accumulation was also restricted to groups of mesophyll Zn-storage cells, whereas it remained low in neighboring non-accumulating ones. Upon exposure to a higher (10 μM) Zn concentration, necrotic regions developed [13], likely originating from the groups of Zn-accumulating cells identified in this study. In contrast, in wild-type tomato Zn was distributed uniformly across mesophyll cells (Fig. 9).

Here we demonstrate that formation of clusters of Zn-accumulating cells in leaves of AtHMA4-tomato plants (Fig. 9) is accompanied by dramatically lower, relative to the wild type, expression of two ethylene receptors *LeNR*, both in the EPP (upper epidermis + palisade parenchyma) and in the ESP (lower epidermis + spongy parenchyma), and *LeETR1* in the ESP (Fig. 7c, g). Lower expression of ethylene receptors in transgenic tomato might indicate fewer receptors within the ER of mesophyll tissues, leading to higher sensitivity to ethylene than in the wild type [41].

These results were the basis for formulating the hypothesis linking the appearance of groups of Zn-accumulating mesophyll cells (Fig. 9) with modifications of the expression profiles of genes from the ethylene biosynthesis pathway detected in transgenic tomato plants exposed to 5 μM Zn (Fig. 7c, g). According to this hypothesis, the combination of higher sensitivity to ethylene than in the wild type with the probably higher production of ethylene (resulting from higher expression of certain *ACO* genes in the mesophyll) could be involved in the pathway signaling Zn excess in the apoplast, leading to induction of Zn accumulation in groups of mesophyll cells. Our previous research on tobacco expressing *AtHMA4* led to the conclusion that until the Zn concentration is sensed as too high, Zn is accumulated uniformly in mesophyll cells, but when the apoplastic Zn reaches a threshold, a signal is generated to redirect Zn to groups of cells (“Zn-storage cells”). The nature of the signal is not known; however, the results of this study suggest that ethylene could be a part of it. These results open a new direction in the search for the mechanisms behind formation of necrotic regions, which, according to our recent data, could be considered a mechanism of tolerance to Zn, protecting neighboring non-accumulating cells from the toxic effects of Zn rather than only being a symptom of toxicity [15]. Future research should clarify this issue. Ethylene is involved in the regulation of numerous physiological processes, including the response to biotic and abiotic stresses [42]. Interestingly, in tomato leaves ethylene accounts for formation of ozone-dependent lesions from specific mesophyll cells, which were considered groups of cells disposed to die upon a certain signal resulting from increased ethylene [43]. Moreover, a recent study on ethylene insensitive mutants etr1-1 and ein2-1 indicated that ethylene signaling is involved in the early Cd stress response in *A. thaliana* leaves [40].

### Expression of *AtHMA4* in tomato modifies expression of cell-wall remodeling genes

Plant cell wall composition and structure play a variety of functions in a plant’s response to metals, including regulation of the capacity for metal accumulation and signaling of metal status [44]. These functions might be distinctly affected in different root and leaf tissues of tomato plants due to overloading of the apoplast with Zn as a result of AtHMA4 export activity.

#### Roots

Expression of *AtHMA4* induced processes leading to strengthening of the cell wall structure by enhanced expression of cell wall structural proteins, extensins, and downregulation of several cell wall-modifying enzymes (Fig. 3d, h). Upregulation of the cell wall structural protein, extensin *uJ-2*, was noted in both root sectors. Stress-induced expression of extensin genes is usually related to the requirement for a fortified cell wall [45]. Significantly higher expression of *uJ-2* in both examined sectors of transgenic roots, with a much higher increase of the transcript level in the EC, indicate hitherto unknown regulation resulting from the high Zn status in the apoplast due to the export activity of the AtHMA4 protein. Increased expression of the cell wall structural gene was accompanied by strong downregulation of expansin-encoding *LeEXP8* and *LeEXP18* genes that facilitate cell wall extension and contribute to cell wall disassembly [46, 47]. Studies have shown that the mRNA of *LeEXP8* accumulates in germinating seeds in the cortical tissue of the root elongation zone [48]. In agreement with this, expression of *LeEXP8* in the wild-type tomato was detected at a high level in the EC only, whereas in the S, the transcript level remained almost undetectable (Fig. 3d). In turn, the expression of *LeEXP18* was detected in aerial parts of young soil-grown seedlings and not in roots [49]. In our experiments, expression of *LeEXP18* decreased to almost zero in transgenics in both sectors, however, in the wild type it was very low in the S, and high in the EC. Thus, this study demonstrates that *LeEXP18* in wild-type tomato is expressed specifically in the EC of the roots, and that a high-Zn apoplastic status in transgenic plants contributes to downregulation of its transcript level. Other downregulated genes in transgenics were plant cell wall-remodeling genes, *THT7-1* and *THT7-8*, that encode an enzyme responsible for the synthesis of hydroxycinnamoyl tyramines. It has been proposed that they promote accumulation of cell wall-bound phenolic amines, and respond to wounding and ozone [50]. It seems that this is an example of common pathways for biotic and abiotic stresses, including the action of heavy metals. Finally, the *XTHs* and *XET* genes involved in modification of the cellulose/xyloglucan network [46, 51] were differentially expressed in transgenic tomato. *XTHs* encodes xyloglucan endotransglucosylase/hydroxylases with two distinct activities. Most act as transglucosylase referred to as XET, while some XTHs, preferentially as hydrolases. The encoded enzymes participate in a range of physiological processes including wall loosening, wall strengthening, cell-wall remodeling during abiotic stress [46, 51]. Interestingly, the accumulation profiles of these genes were different in AtHMA4-tomato, with moderate downregulation of *tXET-B1* in both EC and S root sectors, and very strong upregulation of *XTH3*, specifically in the S. Compared with the wild type, the lower accumulation of *tXET-B1* mRNA in transgenics is in agreement with the overall expression pattern of cell-wall remodeling genes, indicating strengthening of this structure in AtHMA-tomato roots. Transglycosylation by XET can increase or reduce the length of polysaccharides, which could result in either cell wall expansion or disassembly [51].

Expression of the cell wall-remodeling genes was not further analyzed upon longer exposure to the lower (0.5 μM) Zn concentration and in the presence of Cd, as cell wall modification in response to metals was not the major aim of this study. However, these data constitute a basis for further research with a focus on the specific role of the apoplast in a plant’s response to Zn.

#### Leaves

The genes found to be expressed differently in the leaves of transgenic tomato vs. wild type were, except for *LeXTH3,* not the same as those found in roots (Figs. 3 and 7). *LeXTH3* showed higher transcript levels in leaves, specifically in the EPP. In transgenic tomato, in addition to three *XTHs* (*LeXTH1, LeXTH3, LeXTH7*), differential expression of three *Cel* genes encoding endo-1,4-beta-D-glucanases (*LeCel2, LeCel5, LeCel7*) and *LePMEU1* encoding pectin-methyl-esterase (PME) were identified. Endoglucanases are hydrolytic enzymes related to hemicellulose degradation involved in cell wall disassembly during vegetative growth and fruit ripening [46], but also involved in cell wall signaling. Both identified *XTHs* and *Cel* genes are ethylene-inducible [52, 53], and their tissue-specific expression in transgenic plants is accompanied by downregulation of *LeETR1* and *LeNR* receptor genes and upregulation of *ACO* genes, which is very much specific for the EPS and EPP (Fig. 7). In this context, the detected higher expression of *LePMEU1* in transgenic leaves in both EPS and EPP, known from its role in cell wall loosening and cell wall signaling by generation of active oligogalacturonides (OGAs) [54, 55], could be related to signaling the modification of the cell wall structure due to the apoplastic Zn excess. Higher expression of *LePMEU1* was also detected in tomato exposed to 10 μM Zn with enhanced Zn concentration in the apoplast due to expression of *AhHMA4::AhHMA4* [11]. Not much is known about the role of the identified classes of cell wall proteins in plant responses to metals, nonetheless, our study points to their significance in this process. More detailed analysis is needed to demonstrate the nature and physiological significance of these relationships, and to indicate whether they likely contribute to signaling and/or accommodation of Zn excess in the cell wall.

## Conclusions

The major questions asked in this manuscript are: What makes a transgenic plant resistant to changes introduced by a transgene? What endogenous mechanisms are initiated?

The phenomenon of metal-supply-dependent metal root/shoot distribution is known for wild-type plants. It was expected that engineering a desired level of metal accumulation in a target organ by ectopic expression of a chosen metal-homeostasis gene (especially under a strong constitutive promoter) would lead to a modification that remains qualitatively the same under varying conditions of metal supply. It turned out, however, that overcoming the endogenous system controlling the amount of metal transferred to shoots is very difficult, and underlying mechanisms remain unknown [14]. Understanding the regulation of these processes would help to more successfully engineer desired metal accumulation patterns for phytoremediation and biofortification purposes.

In this study, the detected Zn supply-dependent modification of Zn root/shoot distribution in AtHMA4-tomato (increase Zn root-to-shoot translocation at 5 μM Zn, no change at 0.5 μM Zn) involved distinct expression of *LeNRAMP2,* primarily in the stele of the root. Moreover, ethylene-dependent pathways, including ethylene-dependent Fe-uptake system (*LeIRT1, LeFER, LeChln)* and ethylene-related genes (*LeNR, LeACO3, LeACO5*) likely play an important role in this phenomenon. The expression of the identified genes (*LeNR, LeACO3, LeACO5, LeIRT1, LeFER, LeChln*) in transgenic plants was different depending on Zn exposure (upregulated at 0.5 μM Zn, downregulated at 5 μM Zn), and also on Cd exposure.

Ethylene is also suggested as an important factor in a pathway induced by high Zn in the apoplast, which resulted in loading excess Zn into the mesophyll of “Zn accumulating cells”, which might protect the entire leaf blade from Zn toxicity [15]. Furthermore, it was shown that modification of several classes of cell wall-remodeling genes due to overloading of the apoplast with Zn, was organ specific and tissue specific, despite ectopic expression of *AtHMA4*.

Ectopic expression of *AtHMA4* led to deregulation of cellular Zn-status, and induced endogenous metal cross-homeostasis molecular mechanisms in a tissue-specific fashion, likely to counteract these changes. It is noteworthy that more dramatic alterations in the expression level of metal-homeostasis genes were detected in transgenic plants at lower, 0.5 μM, Zn compared with 5 μM Zn, especially in the EC of the roots. Thus, the export activity of AtHMA4 interfered in metal homeostasis to a larger extent at the lower Zn concentration in the medium.

Moreover, the difference in the efficiency of Zn and Cd translocation to shoots between line 4 (higher efficiency) and line 15 was accompanied by differences in the expression of LeNRAMP1-3 (primarily in the EC), with the greatest difference in the expression of *LeNRAMP2* (both in the EC and S).

## Abbreviations

aRNA, amplified RNA; EC, epidermis + cortex; EPP, palisade parenchyma + upper epidermis; ESP, spongy parenchyma + lower epidermis; LCM, laser capture microdissection; RT-qPCR, reverse transcription quantitative polymerase chain reaction; S, stele

## Additional files

Additional file 1: Methodological details on laser microdissection and microarray analysis. (PDF 41 kb) Additional file 2: Tissue embedding for LCM analysis. (PDF 17 kb) Additional file 3: Integrity of RNA isolated from LCM-derived tissues. (PDF 731 kb) Additional file 4: Sequences of primers. (PDF 13 kb) Additional file 5: Reference gene selection for RT-qPCR. (PDF 63 kb) Additional file 6: Zn concentration in roots and leaves. (PDF 21 kb) Additional file 7: Microarray data. (XLSX 2637 kb) Additional file 8: Gene Ontology distribution of gene groups identified in roots (PDF 535 kb) Additional file 9: Selected tomato genes present in microarray. (XLSX 525 kb) Additional file 10: Alteration in transcript levels of selected genes identified in roots by microarrays and verified by RT-qPCR. (PDF 21 kb) Additional file 11: Information on additional genes chosen for expression analysis. (PDF 14 kb) Additional file 12: Gene Ontology distribution of gene groups identified in leaves (PDF 570 kb) Additional file 13: Alteration in transcript levels of selected genes identified in leaves by microarrays and verified by RT-qPCR. (PDF 21 kb) Additional file 14: Alignments of LeNRAMP2 with NRAMPs from other species. (PDF 40 kb)
