# Supplementary material for: Engineering high Zn in tomato shoots through expression of AtHMA4 involves tissue-specific modification of endogenous genes
Source: BMC Genomics. 2016 Aug 12;17:625. doi: 10.1186/s12864-016-2990-x (PMC4982198; doi:10.1186/s12864-016-2990-x)
Supplement: Additional file 1: — Methodological details on laser microdissection and microarray analysis. (PDF 41 kb) [file 12864_2016_2990_MOESM1_ESM.pdf]

Additional file 1: Methodological details on laser microdissection and microarray analysis.

Cryosectioning, laser microdissection, and microarray

Cryosectioning

Apical fragments of roots, 15 mm long, were cut off and subsequent 2 cm-long segments were collected. From leaves (the 3rd and 4th leaf counting upwards), 1–2 mm-long and 2–3 mm-wide fragments were cut out from between the vascular bundles in the middle of each leaf.

The collected tissues were fixed according to a modified procedure described by [1]. Briefly, the root or leaf fragments were infiltrated on ice with a mixture of glacial acetic acid:ethanol (1:3 v/v) for 10 min under 0.5 Ba, followed by infiltration with PBS containing 30% sucrose (conditions as above). Thus prepared root or leaf fragments were placed in sippers (RNaseZap-treated and washed with DEPC-treated water) filled with NEG-50 Frozen Section Medium (Thermo Scientific), and frozen in liquid nitrogen. Each sipper contained 20-30 root fragments or 6-8 leaf fragments. The samples were sectioned immediately or stored at –80 °C for up to 10 days.

Sectioning was done in a HM 560 cryostat (Microm) at –20 °C. Sections 14 μm thick were cut and mounted on poly-L-lysine-coated slides (RNaseZap-treated, washed with DEPC-treated water and air-dried). In order to dehydrate the tissue, the slides were immersed for 10–30 seconds in Tissue-Tek® Tissue-Clear® Xylene Substitute (Sakura) followed by 100% ethanol, both chilled to –20 °C. Directly before laser microdissection, the slides were removed from the cryostat chamber and air-dried at room temperature.

Laser capture microdissection (LCM)

The PALM Microlaser System (P.A.L.M. Microlaser Technologies http://www.zeiss.de/microdissection) was used to isolate the (i) epidermis+cortex (EC) and stele (S) from root sections; (ii) palisade parenchyma+upper epidermis (EPP) and spongy parenchyma+lower epidermis (ESP) from leaf sections. This LCM system consists of a 337 nm pulsed nitrogen laser coupled to an inverted microscope. The energy and focus settings of the UV-laser were adjusted to allow cutting and collecting the tissue without causing its charring. The fragments of interest were selected, dissected via UV-laser excision, and catapulted onto the lid of a 0.5 ml Eppendorf tube filled with 30 μl of PicoPure RNA

extraction buffer (Arcturus Engineering, CA). Each tube contained about 100 tissue fragments isolated in the LCM procedure. Three technical replicates were collected for each tissue type. Right after collection, the samples were incubated at 42 °C for 30 min and centrifuged for 2 min at 800 x g, RT (first stage of RNA extraction). At this point the samples were stored at – 80 °C until further processing.

Since the PALM LCM system uses laser impulses to catapult tiny fragments of tissue, it was not possible to microscopically assess contamination. During LCM of leaf tissue we left a narrow margin of uncollected tissue on the border between spongy parenchyma and palisade parenchyma to avoid cross-contamination. Moreover, from each leaf section only one tissue was collected. In case of roots, tissue cross-contamination was verified using real-time PCR. We checked transcript levels for *LeIRT1* which is known to be expressed only in the epidermis+cortex. In our study we used only those samples of stele that showed no detectable transcript of this gene. The Arcturus XT LCM system used later in our study allows collection of large areas of intact tissue (in our case, the whole stele or epidermis+cortex from a root cross section) on a transparent adhesive cap. This allowed us to make a preliminary assessment of collected tissue fragments under a microscope.

RNA extraction and amplification

RNA was isolated using the PicoPure RNA Isolation Kit following the manufacturer's instructions, including treatment with the RNase-Free DNase Set (Qiagen, USA). For each tissue, three technical replicates were pooled together giving a total volume of 90 μl. Due to a greater initial volume, 90 μl of 70% ethanol was added to each sample and RNA binding to the column was conducted for 5 min at 100 × g. The RNA was eluted from the purification column with 14 μl of low-ionic-strength elution buffer.

The RNA was amplified with the RiboAmp HS PLUS (Arcturus Engineering) amplification system following the manufacturer's protocol except for DNase treatment. Instead of the DNase provided in the RiboAmp HS PLUS kit, the RNase-Free DNase Set (Qiagen, USA) was used. Directly after in vitro transcription (IVT), 4 μl of DNase (1 μl of DNase diluted in 4 μl RDD buffer) was added to the samples and incubated for 15 min at RT. Immediately after DNase treatment, the amplified RNA (aRNA) purification stage was performed following the manufacturer's recommendations. Two rounds of amplification were required to generate a sufficient amount of aRNA.

The RNA concentration in samples was quantified at A_{260} using a Nanodrop spectrophotometer ND 2000 (Nanodrop, Wilimington, USA). RNA integrity was assessed at each step of isolation and amplification in an Agilent 2100 Bioanalyzer apparatus (Agilent Technologies) using Agilent RNA 6000 Pico reagents (Agilent Technologies). RNA samples were chosen for amplification on the basis of RIN (above 6 for root tissues or 4 for leaf tissues, for more information in Supplementary Info S1), as well as visual evaluation of band pattern and hints of degradation on electropherograms. The samples were stored at –80 °C.

Microarray transcriptome analysis

To determine whether ectopic expression of *AtHMA4* modifies the tissue-specific gene expression pattern in tomato roots and leaves, microarray analysis was performed. aRNA from root and leaf tissues of transgenic line 4 and wild-type tomato grown in the presence of 5 μM Zn was used. Samples with high aRNA concentrations and comparable quality were selected for microarray analysis and diluted with nuclease-free sterile water (Ambion) to a final concentration of 10 ng/μl in a volume of 10 μl. The analysis was performed with three independent biological replicates.

The microarray experiment was carried out at Corelab, Laboratory of Microarray Analyses (University of Warsaw and Institute of Biochemistry and Biophysics, PAS). GeneChip Tomato Genome Array (Affymetrix) microarrays consisting of over 10 000 *L. esculentum* probe sets were used. aRNA synthesis with a GeneChip 3′ IVT - Express Kit (Affymetrix) using 100 ng of mRNA was carried out according to the manufacturer’s instructions. Hybridization and labeling were performed using GeneChip® Hybridization, Wash, and Stain Kit (Affymetrix) following the manufacturer’s protocol. The microarrays were scanned with a GeneChip® Scanner 3000 7G.

Microarray Data Analysis

The microarrays were scanned with an Attfymetrix GeneChip Scanner, and the intensity signals for each probe set were written by Affymetrix GeneChip® Command Console® (AGCC) software into CEL files. Pearson's analysis was applied in order to estimate the correlation between linear variables and, therefore, to examine the homogeneity of the samples. The CEL files were imported into Partek Genomic Suite v 6.6 software with the use of GCRMA (GC Robust Multiarray Averaging). During this step background correction was applied based on the global distribution of the PM (perfect match) probe intensities and the affinity for each of the probes (based on their sequences) was calculated.

Further, the probe intensities were quantile normalized [2], log2 transformed and median polish summarization to each of the probe sets was applied. Qualitative analysis was performed next. Principal Component Analysis (PCA) was used to identify outliers and artifacts on the microarray. After the quality check, 3-way Analysis of Variance (ANOVA) with restricted maximum likelihood estimation (REML) [3] was performed on the data, which allowed creating lists of significantly and differentially expressed genes between the biological variants (with the cutoff values: p-value < 0.05, -1.2 > Fold Change > 1.2).

Transcript profiles were compared between transgenic line 4 and wild type in the (i) epidermis+cortex (EC) and in stele (S) of roots; (ii) upper epidermis+palisade parenchyma and lower epidermis+spongy parenchyma of leaves. A similar comparison was performed between the epidermis+cortex and stele of roots, as well as between the lower epidermis+spongy parenchyma (ESP) and upper epidermis+palisade parenchyma (EPP) of leaves. Two-way ANOVA was performed and genes with a false discovery rate (FDR) of < 0.05 were considered significantly altered in their expression in the transformants compared with the wild type or between the tissue types. The gene lists were then filtered to select those with a fold change > 1.2. Since the *Affymetrix® GeneChip® Command Console®* Software uses an obsolete tomato genome database, the obtained gene lists were verified by searching the Tomato Functional Genomics Database (http://ted.bti.cornell.edu/cgi-bin/TFGD/array/home.cgi) containing an updated base of tomato microarray probes. Functional categorization was performed using Gene Ontology (GO) analysis tools available at Tomato Functional Genomics Database (http://ted.bti.cornell.edu/cgi-bin/TFGD/array/home.cgi).

Genes of major interest differentially expressed in transformants relative to wild type were identified and chosen for quantitative real-time PCR (qRT-PCR) analysis to confirm detected differences. For that purpose, plants grown under the same conditions as those in the microarray experiment were used. Three gene categories were employed: (1) metal transport/homeostasis; (2) ethylene biosynthesis (reports indicate a link between ethylene biosynthesis and regulation of the expression of certain metal transport genes); (3) cell wall modifications (the cell wall is a compartment involved in a plant's response to metals).
The available microarrays are far from being complete when it comes to characterization of tomato genes. While the probe set used in the study represents the better known (although frequently not fully understood) fraction, the overall depth of gene annotation in tomato is not sufficient. When we deleted the genes present in probes from the Unigene database of transcribed tomato loci (over 18000 records), the resulting list of identifiers could have been

mapped to 2947 Uniprot proteins (only 30 have the status "reviewed"), compared with the 4218 proteins (with 230 with "reviewed" status) for the Unigene records that are present in the microarray. The genes from four categories present in the microarray, that are in focus in this research (1. metal transport; 2. transcription factors; 3. ethylene biosynthesis; 4. cell wall modifications) are listed in Table S2.

References:

[1] Bolstad BM, Irizarry RA, Astrand M, Speed TP. A comparison of normalization methods for high density oligonucleotide array data based on variance and bias. Bioinformatics 2003; 19: 185-193.

[2] Nakazono M, Qiu F, Borsuk LS, Schnable PS. Laser-Capture Microdissection, a Tool for the Global Analysis of Gene Expression in Specific Plant Cell Types: Identification of Genes Expressed Differentially in Epidermal Cells or Vascular Tissues of Maize, Plant Cell 2003; 15: 583-96.

[3] Thompson WA. The problem of negative estimates of variance components. Annals Mathem. Stat. 1962; 33: 273-289.
