# Supplementary material for: Engineering high Zn in tomato shoots through expression of AtHMA4 involves tissue-specific modification of endogenous genes
Source: BMC Genomics. 2016 Aug 12;17:625. doi: 10.1186/s12864-016-2990-x (PMC4982198; doi:10.1186/s12864-016-2990-x)
Supplement: Additional file 2: — Tissue embedding for LCM analysis. (PDF 17 kb) [file 12864_2016_2990_MOESM2_ESM.pdf]

Additional file 2: Tissue embedding for LCM analysis

Analytical procedure for tissue fixation, embedding, and sectioning for LCM analysis

Tissue fixation and sectioning

Root apical fragments 15 mm long were cut off, and the subsequent 2 cm-long segments were fixed in pre-chilled methanol:acetic acid (3:1 v:v) overnight (approx. 16 hours) at –20 ^{0}C (modified method described by Kozubek [1]. Fixed roots were dehydrated in glass vials at 4 ^{0}C in a graded methanol series (75%, 85%, 95%, 100%; 30 min each), followed by a mixture of methanol:ethanol (3:1, 1:1, 1:3; v:v; 1h each), then in 100% ethanol three times for 1 h. Tissues were then embedded in Steedman's wax [2,3]. Blocks were formed at room temperature and stored at +4 ^{0}C in a closed container containing a moisture absorber.

Sectioning was performed at –5 ^{0}C in an HM 560 cryostat (Microm). The cryostat knife was autoclaved twice prior to use and the entire cryostat chamber was treated with RNaseZap. Blocks were affixed to a holder with a few drops of liquid Steedman's wax and treated with RNaseZap directly before sectioning. Sections (14 µM) were affixed to PEN-membrane slides (RNaseZap treated, rinsed with nuclease free water and air-dried) and stretched with a few drops of nuclease free water (Ambion). The specimens were dried at 32 ^{0}C overnight. Directly before laser capture microdissection, the sections were de-waxed by submerging in 100% ethanol for about 30 minutes. In order to remove the wax thoroughly, the alcohol was replaced three times.

Laser capture microdissection (LCM)

The ArcturusXT™ LCM System (Arcturus Engineering, CA) was used to isolate the epidermis+cortex (EC) and stele (S) from root sections. This LCM system consists of an 810 nm IR Capture Laser and a 355 nm UV Cutting Laser coupled to a Nikon Eclipse® Ti-E microscope. Prior to the LCM procedure, the stage of the microscope as well as the surrounding work station were treated with an antistatic spray, Ania (Barwa), and RNaseZap to prevent RNA degradation and sample contamination. The energy and focus settings of the IR-laser were adjusted to allow collecting the tissue without damaging it. The fragments of interest were selected, attached to the adhesive membrane of CapSure® Macro LCM Caps (Arcturus Engineering, CA) via IR laser and cut via UV-laser. After the cap was completely covered with tissue fragments, it was placed in a 0.5 ml Eppendorf tube filled with 30 µl of PicoPure RNA extraction buffer (Arcturus Engineering, CA). Three technical replicates were collected for each tissue type. Right after collection, the samples were incubated at 42 ^{0}C for

30 min and centrifuged for 2 min at 800 x g, RT (first stage of RNA extraction). The samples were stored at –80 ^{0}C for further RT-PCR analysis.

References:

[1] Kozubek S, Lukášová E, Amrichová J, Kozubek M, Lišková A, Šlotová J. Influence of Cell Fixation on Chromatin Topography. Anal. Biochem. 2000; 282: 29–38.

[2] Norenburg JL, Barrett JM. Steedman's polyester wax embedment and de-embedment for combined light and scanning electron microscopy. J. Electr. Microsc. Techn. 1987; 6: 35-41.

[3] Gomez SK, Javot H, Deewatthanawong P, Torres-Jerez I, Tang Y, Blancaflor EB, Udvardi MK, Harrison MJ. *Medicago truncatula* and *Glomus intraradices* gene expression in cortical cells harboring arbuscules in the arbuscular mycorrhizal symbiosis. BMC Plant Biol. 2009; 9: 10.
