# Supplementary material for: Engineering high Zn in tomato shoots through expression of AtHMA4 involves tissue-specific modification of endogenous genes
Source: BMC Genomics. 2016 Aug 12;17:625. doi: 10.1186/s12864-016-2990-x (PMC4982198; doi:10.1186/s12864-016-2990-x)
Supplement: Additional file 3: — Integrity of RNA isolated from LCM-derived tissues. (PDF 731 kb) [file 12864_2016_2990_MOESM3_ESM.pdf]

Additional file 3: Integrity of RNA isolated from LCM-derived tissues from frozen and Steedman's wax-embedded roots and leaves

Two protocols of tissue preparation for sectioning for subsequent laser microdissection were used: (i) snap freezing; (ii) embedding in Steedman's wax. In both cases, the quality of isolated RNA was high with distinct bands representing the 28S and 18S rRNA molecules. The RNA integrity number (RIN) indicating the quality of the RNA samples was determined for each RNA sample isolated from each round of laser microdissected tissues (Supplementary Info 3A – lower in this file). The RNA integrity number (RIN) is a software algorithm designed to estimate the integrity of total RNA samples. It assigns RNA samples a score of 1 to 10, where 10 is completely intact and 1 is totally degraded. Only RNA samples with an RIN above six (from roots) or above four (from leaves – explanation below) were used for analysis.

RNA samples of good quality from the same experimental trial and tissue were pooled. Such pooled RNA samples were subjected to amplification before being used for cDNA synthesis. To obtain enough RNA (each sample of isolated cell sections generated only 3–60 ng total RNA), two rounds of amplification were performed. Samples with the highest aRNA content and similar quality were divided. One part was diluted and used for microarray-based global transcription profile analysis; the second was used for real-time RT-PCR expression study. Representative electropherograms and corresponding images of the gel after electrophoresis are shown in Suoolementary Info 3B (down in this file). Two clear bands of 18S rRNA and 28s rRNA appeared on the gel image of root RNA and the RIN reached six and above. On electropherograms of leaf RNA, five bands of plastidic RNA were clearly visible in addition to 18S rRNA and 28s rRNA. The RIN algorithm does not include any additional bands, therefore, its value was lower for tomato leaf RNA. In this case, samples with RIN reaching four and displaying an appropriate band pattern without visual traces of degradation on electropherograms were chosen for further analysis.

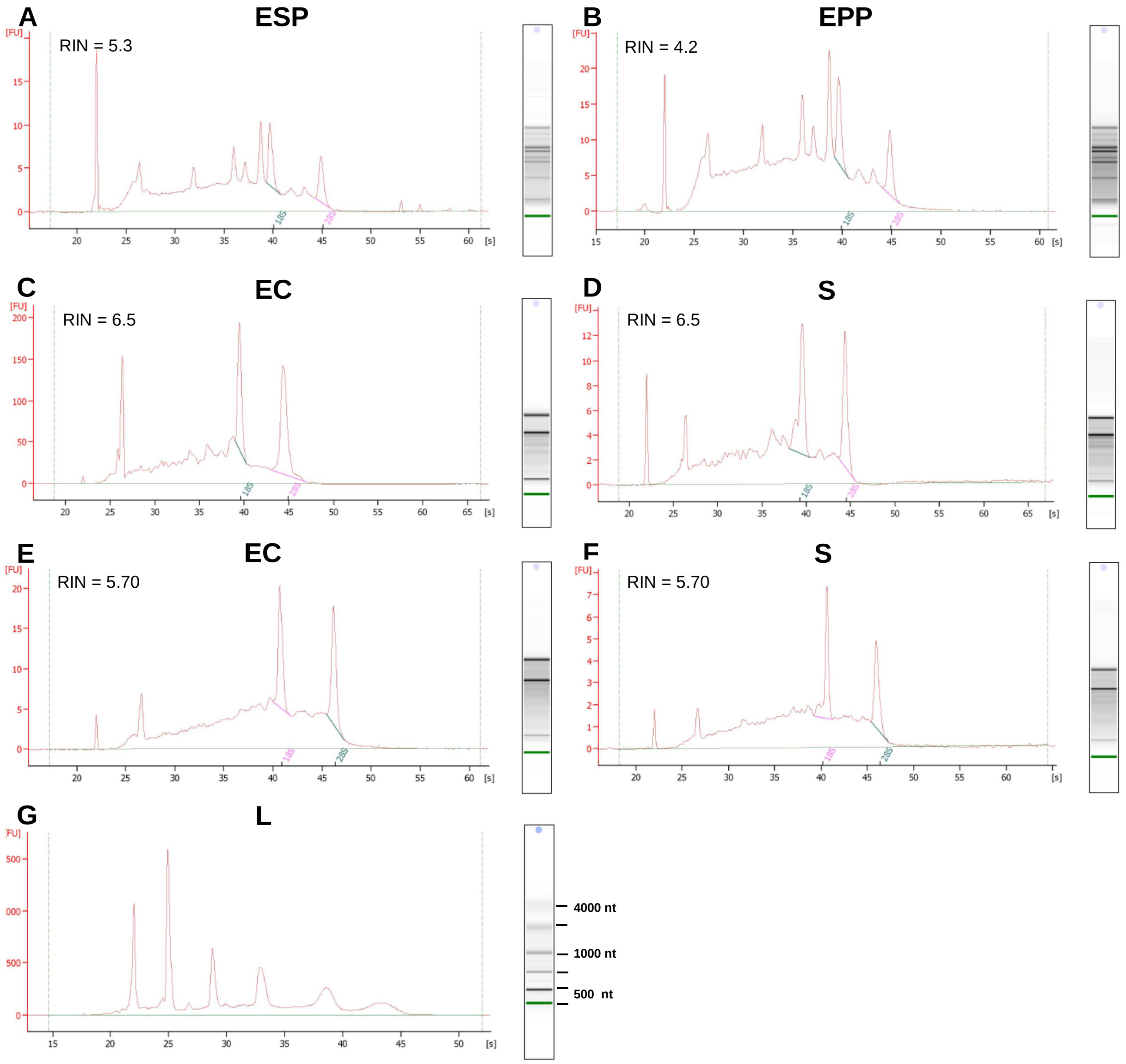

3A. Quality control of the integrity of LCM-derived total RNA from AtHMA4-expressing tomato line 4 tissues. Electropherogram and gel depiction of RNA isolated from (A) spongy paerenchyma + lower epidermis (ESP); (B) palisade paerenchyma + lower epidermis (EPP) derived from the sections of frozen leaf fragments; (C) epidermis +cortex (EC); (D) stele (S) derived from the sections of frozen root fragments (E) epidermis +cortex (EC); (F) stele (S) derived from the sections of methanol:acetic acid (3:1 v/v) fixed Steedman's wax embedded root fragments; (G) RNA ladder (L).

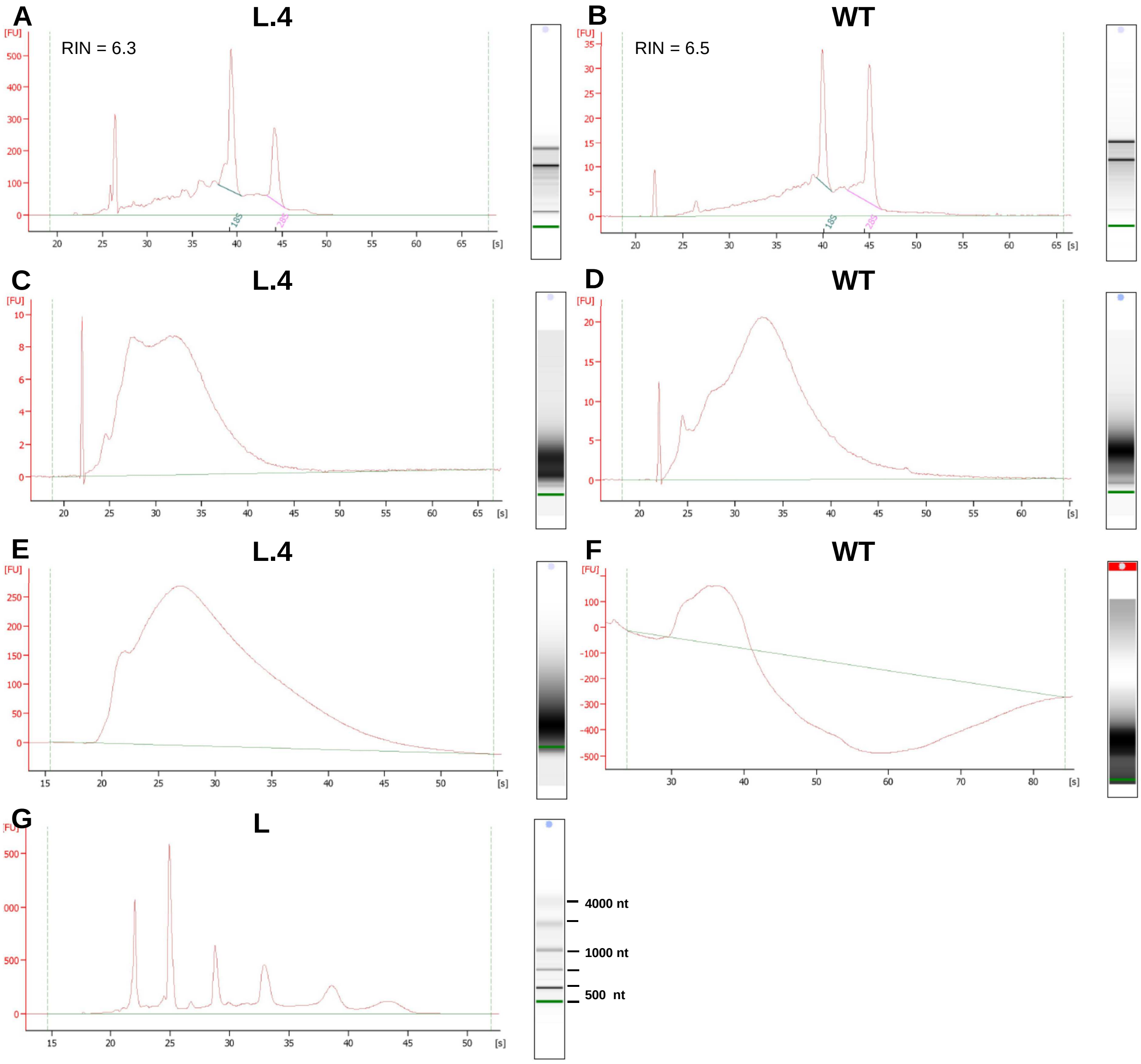

3B Visualization of LCM-derived RNA amplification by electropherogram and gel depiction obtained using bioanalyzer measurements RNA Quality control of RNA from epidermis+cortex of AtHMA4 transformant (L.4) and wild-type tomato; (A, B) total RNA; (C, D) aRNA after first round of amplification; (E, F) aRNA after second round of amlpification; (G) RNA ladder
