# Supplementary material for: Engineering high Zn in tomato shoots through expression of AtHMA4 involves tissue-specific modification of endogenous genes
Source: BMC Genomics. 2016 Aug 12;17:625. doi: 10.1186/s12864-016-2990-x (PMC4982198; doi:10.1186/s12864-016-2990-x)
Supplement: Additional file 4: — Sequences of primers. (PDF 13 kb) [file 12864_2016_2990_MOESM4_ESM.pdf]

Additional file 4. **Sequences of primers used for expression analysis.**

Gene	NCBI accession number	Primer sequences	Product size
NRAMP1	AY196091	F: CACTCTGCCCTTGTACTATC<br>R: GTACCAGACACAGACACAAC	142
NRAMP2	AY562196	F: GGAGCTTTTAGCAGGTCTCT<br>R: GATCTCTCTGGATTGCACCA	133
NRAMP3	AY196092	F: TGTTTGGAGAGACGAAACCA<br>R: ACACCCTACAATTCCCACTG	104
IRT1	AF136579	F: TCCTACAGGCGGAGTATAAG<br>R: CAGTTATTAACGCCCGTGGA	138
FRO1	AY224079	F: CTCTACCAGATGATCTCCTCC<br>R: GAGTAATTCCACTTCCTCCAC	135
NbABC	AY943856	F: TCCACTGTGCAGAACTGGTATG<br>R: TGACCCTGTCTCCACTTGTG	129
Chln	AJ242045	F: GGTGCCTATGTGTTACCTAG<br>R: CTCACTCACTCCTCCAGAG	106
FER	AF437878	F: GCAACAAAG GGCGACACATTG<br>R: CCAACGGAGATGTCTCGAAGTC	228
bZIP44	FJ647190	F: ACAGTGGACTTGTCATGGACC<br>R: TGCAAGACATCGGCAGTC	141
NR	U38666	F: GCTCGATGTAAAAATCCGATGG<br>R: CACTGAACTACTACTCCACG	101
ETR1	NM_001247220	F: TCTAAGCTCCCCTTTGTGAC<br>R: CATCCAAGGTGTGTAAGCAG	134
ACO1	NM_001247095	F: CTAACGGGAAGTACAAGAGTGTG<br>R: TCCTCTGCCTCTTTTTCAACC	151
ACO3 (E8)	XM_004247652	F: GCTGCAAGTTGGAGAGACAC<br>R: CCTCTGCCACTGAGCTACT	134
ACO4	NM_001246999	F: TGACGCGGTGATCTATCCA<br>R: CCACATTAGCTTCCATAGCCT	158
ACO5	AJ715790	F: TCTTTGTTTGGCATCGTCCA<br>R: CTAGGCCAAGGTTCTCACAC	147
ACO6	NM_001247709	F: GCTCACACAGATGCTGGTG<br>R: GAGTTGGTCACCGAGGTTG	138
PMEU1	U70677	F: GTGGCCGGAGTGGTTGTCCG<br>R: CGCAGAGTGCCACGGCTTGA	395
EXP8	AF184232	F: GGAGGTGCTTGTGGATATG<br>R: CCTTTTTGCACCATTGAGG	154
EXP18	AJ004997	F: GTTGGAGGTGAAAGGGGAAG<br>R: CGGGCTACATGTTTTTGCAG	118
uJ-2	X55686	F: GGAACCAAAACCATCACCAC<br>R: CGTAGGAGACTCCGATTACC	197
THT7-1	AY081906	F: CTCAACAACCAACTCCATCTC<br>R: TCAAGTAGA AGTACGGATGG	264
THT7-8	AY081907	F: GCATCATCGGAAAACAACAAC<br>R: TCAAGTAGA AGTACGGATGG	223
XTH1	NM_001246929	F: TGAGGCTTGTTGGTGGAGAC<br>R: CTCTGTTTCCTTTTCCTCCTGTG	162

XTH3	NM_001247543	F: GTCATCACAAGGACCAACAC<br>R: TGGAAATCAGCAGTAGGGTC	156
XTH7	NM_001245746	F: GGAACCATCATCAAGCCGTG<br>R: CTCTTGTAGCCCAGTCGTCAG	147
tXET-B1	X82685	F: CTATGTCCTGTTTGGGCTGA<br>R: CAAGGGACAAGGCGAGAAG	106
Cel 2<br>*AAA69909*	U13055	F: GAAAGCTACGGCGAGAGATGG<br>R: ACATCGGAACCGGGATTATTCG	141
Cel 5<br>*AAC62241*	U78526	F: GGTTCTGATGTTGCTGCTGAG<br>R: GCAGACCACTGAACTGAGACTG	171
Cel 7<br>*CAA72133*	AF308936	F: GAGCTATATTGGGGAGCAGC<br>R: GTCATTTCCTACCACGCTTCTCC	168
CyP	NM_001247559	F: CTCTTCGCCGATACCACTCC<br>R: TCACACGGTGGAAGGTTGAG	120
