# Supplementary material for: Engineering high Zn in tomato shoots through expression of AtHMA4 involves tissue-specific modification of endogenous genes
Source: BMC Genomics. 2016 Aug 12;17:625. doi: 10.1186/s12864-016-2990-x (PMC4982198; doi:10.1186/s12864-016-2990-x)
Supplement: Additional file 5: — Reference gene selection for RT-qPCR. (PDF 63 kb) [file 12864_2016_2990_MOESM5_ESM.pdf]

Additional file 5: Reference gene selection for RT-qPCR gene transcription studies. (a) microarray identification of differential expression of genes commonly used as references in **root** and **leaf** tissues of AtHMA4- expressing (L.4) and wild-type 17-day-old tomato plants grown in the presence of 5 µM Zn; (b) transcription level of actin (ACT), tubulin (TUB), and cyclophilin (CyP) in the epidermis+cortex and stele of roots from AtHMA4- expressing (L.4) and wild type 24-day-old tomato plants grown in the presence of 5 µM Zn; (c) Relative expression of cyclophilin in the epidermis+cortex and stele of roots from AtHMA4- expressing (L.4 and L.15) and wild-type 24-day-old tomato plants grown in the presence of 0.5 µM Zn; (d) Relative expression of cyclophilin in epidermis+cortex and stele of roots from AtHMA4-expressing (L.4 and L.15) and wild type 24-day-old tomato plants grown in the presence of 1 µM Cd; (e) Relative expression of cyclophilin in palisade parenchyma + upper epidermis and spongy parenchyma + lower epidermis of leaves from AtHMA4- expressing (L.4 and L.15) and wild type 17-day-old tomato plants grown in the presence of 5 µM Zn. The relative expression level was calculated for 172 pg of cDNA template used in the reaction. All accessions numbers are from NCBI.

(a)

Leaves

L.4 vs WT EPP		L.4 vs WT ESP		L.4 ESP vs EPP		WT ESP vs EPP	
Gene	Fold- Change	Gen	Fold- Change	Gene	Fold- Change	Gene	Fold- Change
ABB13293 beta-tubulin [Lycopersicon esculentum]	-1,70126	ABB13293 beta-tubulin [Lycopersicon esculentum]	-2,40579	ABB13293 beta-tubulin [Lycopersicon esculentum]	-1,78593	ABB13293 beta-tubulin [Lycopersicon esculentum]	-1,26293
AAB51592 glyceraldehyde 3-phosphate dehydrogenase [Lycopersicon esculentum]	1,64479			AAB51592 glyceraldehyde 3-phosphate dehydrogenase [Lycopersicon esculentum]	2,24799	AAB51592 glyceraldehyde 3-phosphate dehydrogenase [Lycopersicon esculentum]	4,24621
CAA51679 ubiquitin [Solanum lycopersicum]	-1,30168			CAA51679 ubiquitin [Solanum lycopersicum]	1,75594	CAA51679 ubiquitin [Solanum lycopersicum]	1,413
		P17786 RecName: Full=Elongation factor 1-alpha; Short=EF-1-alpha	1,2014	P17786 RecName: Full=Elongation factor 1-alpha; Short=EF-1-alpha	1,30828		
				CAA41207 ubiquitin [Solanum lycopersicum]	1,42027		
				P17786 RecName: Full=Elongation factor 1-alpha; Short=EF-1-alpha	1,20743		
				P17786 RecName: Full=Elongation factor 1-alpha; Short=EF-1-alpha	1,20216		
		P17786 RecName: Full=Elongation factor 1-alpha; Short=EF-1-alpha	-1,3563	P17786 RecName: Full=Elongation factor 1-alpha; Short=EF-1-alpha	-1,35314		
						AAK95390 ribosomal protein L2 [Lycopersicon esculentum]	1,36971

Roots

L.4 vs WT EC		L.4 vs WT S		L.4 EC vs S		WT EC vs S	
Gen	Fold- Change	Gen	Fold- Change	Gen	Fold- Change	Gen	Fold- Change
AAB51592 glyceraldehyde 3-phosphate dehydrogenase [Lycopersicon esculentum]	-2,27524			AAB51592 glyceraldehyde 3-phosphate dehydrogenase [Lycopersicon esculentum]	-2.01383		

(b)

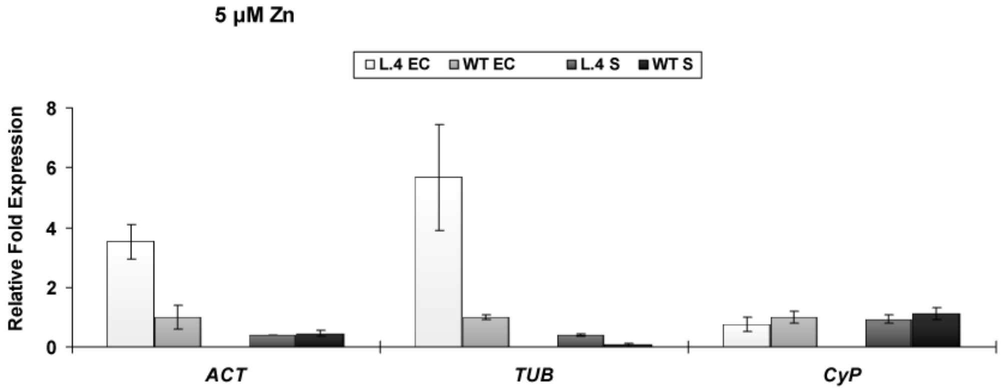

5 µM Zn
L.4 EC
WT EC
L.4 S
WT S
Relative Fold Expression
8
6
4
2
0
ACT
TUB
CyP

(c)

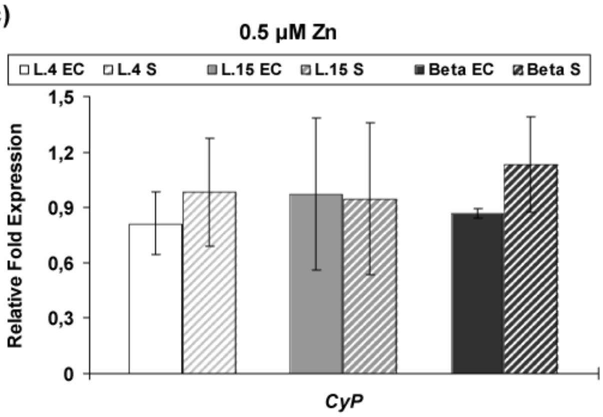

0.5 µM Zn
L.4 EC
L.4 S
L.15 EC
L.15 S
Beta EC
Beta S
Relative Fold Expression
1,5
1,2
0,9
0,6
0,3
0
CyP

(d)

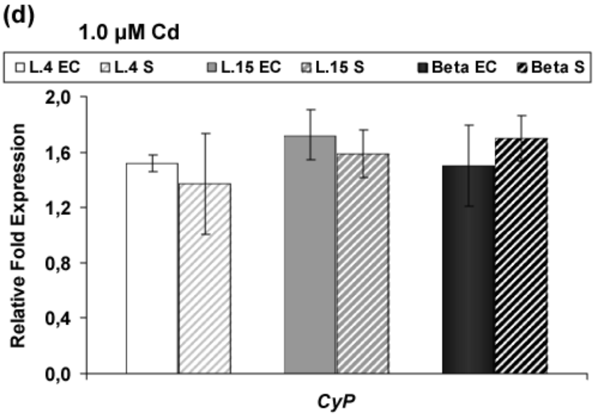

1.0 µM Cd
L.4 EC
L.4 S
L.15 EC
L.15 S
Beta EC
Beta S
Relative Fold Expression
2,0
1,6
1,2
0,8
0,4
0,0
CyP

(e)

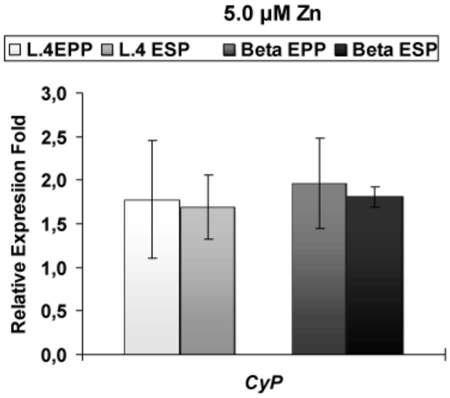

5.0 µM Zn
L.4EPP
L.4 ESP
Beta EPP
Beta ESP
Relative Expresiion Fold
3,0
2,5
2,0
1,5
1,0
0,5
0,0
CyP
