# Supplementary material for: Engineering high Zn in tomato shoots through expression of AtHMA4 involves tissue-specific modification of endogenous genes
Source: BMC Genomics. 2016 Aug 12;17:625. doi: 10.1186/s12864-016-2990-x (PMC4982198; doi:10.1186/s12864-016-2990-x)
Supplement: Additional file 6: — Zn concentration in roots and leaves. (PDF 21 kb) [file 12864_2016_2990_MOESM6_ESM.pdf]

Additional file 6: Zn concentration in roots and leaves .

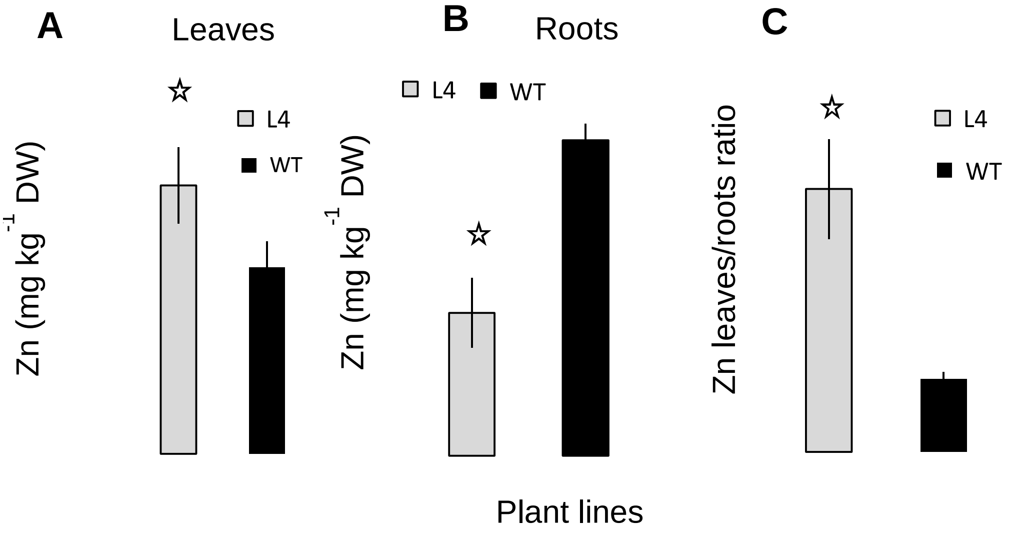

Zn concentration in 24-day old transgenic (line 4) and wild-type (WT) plants exposed to 5 µM Zn for 2 weeks. Zn concentration in leaves (A); roots (B); leaves/roots Zn, concentration ratio (C). Values correspond to arithmetic means ± SD (n=6); values significantly different from WT are highlighted by an asterisk (P≤0.05) (evaluated by Student's *t*-test).
