# Supplementary material for: Engineering high Zn in tomato shoots through expression of AtHMA4 involves tissue-specific modification of endogenous genes
Source: BMC Genomics. 2016 Aug 12;17:625. doi: 10.1186/s12864-016-2990-x (PMC4982198; doi:10.1186/s12864-016-2990-x)
Supplement: Additional file 10: — Alteration in transcript levels of selected genes identified in roots by microarrays and verified by RT-qPCR. (PDF 21 kb) [file 12864_2016_2990_MOESM10_ESM.pdf]

Additional file 10: Alteration in transcript levels of selected genes identified in roots by microarrays and verified by RT-qPCR. Changes in expression levels in epidermis+ cortex (EC) and stele (S) of roots of 17-day old transgenic plants line 4 versus wild type Beta grown for one week in the presence of 5 µM Zn.

↑ **-** upregulated**,** ↓ **-** downregulated, “blank” - similar transcript level

In cases where transcripts were detected by Real-time PCR in one tissue type only, fold change could not be calculated. The results are then shown as arrows indicating the course of change.

All accessions numbers are from NCBI.

**Accession nuber**	**Gene product**	**Microarray**				**RT-qPCR**	
		**EC**		**S**		**EC**	**S**
		**Fold change**	**p-value**	**Fold change**	**p-value**	**Fold change**	**Fold change**
Metal transporters and uptake facilitators:							
AY562196	Natural resistance-associated macrophage protein 2 (*NRAMP2*)	↑ 1.5	0.0185853			↓	
AY943856	ABC putative transporter (*Nicotiana benthemiana*)*	↓ -2.5	0.0355472			↓	↓
Transcription factors:							
AF437878	Basic helix-loop-helix (bHLH) transcriptional regulator (*FER*)	↑ 7.7	0.0336576	↑ 19.5	0.00422614	↓	↓
FJ647190	Basic region/leucine zipper motif transcription factor 44 (*bZIP44)*			↑ 3.1	0.00929777		
Ethylene pathway:							
U38666	Never-ripe ethylene receptor (*Never-ripe*)			↓ -2.0	0.000258931	↓	↓
XM_004247652	1-Aminocyclopropane-1-carboxylate oxidase 3 (*ACO3*, *E8*)	↓ -5.4	0.0118337			↓	
NM_001246999	1-Aminocyclopropane-1-carboxylate oxidase 4 (*ACO4*)	↓ -14.6	0.01920950			↓	↓
AJ715790	1-Aminocyclopropane-1-carboxylate oxidase5 (*ACO5*)	↓ -235.9	0.00628689	↓ -185.3	0.00460845	↓	↓
Cell wall modifications:							

AF184232	Expansin 8 (*Exp 8*)	↑ 15.7	0.0446484			↓	↓
AJ004997	Expansin 18 (*Exp 18*)	↓ -2.2	0.0309869			↓	↓
X55686	Extensin class II (*uJ-2*)**	↑ 13.1	0.0374194			↑	↑
AY081906	N-hydroxycinnamoyl-CoA:tyramine N-hydroxycinnamoyl transferase (*THT7-1*)	↓ -14.5	0.0287053			↓	↓
AY081907	N-hydroxycinnamoyl-CoA:tyramine N-hydroxycinnamoyl transferase 7-8 (*THT7-8*)	↓ -7.0	0.0203166			↓	↓
NM_001247543	Xyloglucan endotransglucosylase/hydroxylase 3 (*XTH3*)			↓ -3.8	0.0201932		↑
X82685	Xyloglucan endotransglucosylase (*tXET-B1)*			↓ -8.6	0.037893	↓	↓

* probe no. LesAffx.65412.1.S1_at best hit: non-intrinsic ABC protein from *Nicotiana benthamiana*; target sequence best hit: UPF0051 protein ABCI8, max identity 98%

** Extensin class II clone uJ-2 characterised by Showalter [1]

[1] A.M. Showalter, J. Zhou, D. Rumeau, S.G. Worst, J.E. Varner, Tomato extensin and extensin-like cDNAs: structure and expression in response to wounding. Plant Mol. Biol. 16 (1991) 547-565.
