# Supplementary material for: Engineering high Zn in tomato shoots through expression of AtHMA4 involves tissue-specific modification of endogenous genes
Source: BMC Genomics. 2016 Aug 12;17:625. doi: 10.1186/s12864-016-2990-x (PMC4982198; doi:10.1186/s12864-016-2990-x)
Supplement: Additional file 11: — Information on additional genes chosen for expression analysis. (PDF 14 kb) [file 12864_2016_2990_MOESM11_ESM.pdf]

Additional file 11: Information on additional genes chosen for expression analysis.

IRT1 (probe ID: Les.3504.1.S1_at) : Expressed only in roots. Fold change was < 1.2 and *P*-value exceeded 0.05 considerably so it was not taken into account in microarray results. However, the IRT1 probe hybridization signal was low, between 2.4 and 3.1 (table S1), therefore, we decided to include it in the RT-qPCR study.

NRAMP1 (probe ID: Les.4303.1.S1_at): Expressed only in roots. Despite the fold change > 1.2, the *P*-value was too high (> 0.05) to be considered in microarray results, nonetheless, we included it in the RT-qPCR experiment.

NRAMP3 **(**probe ID: Les.2567.1.S1_at): In leaves identified as differently expressed in *AtHMA4*-expressing tomato than in the wild type. In the stele of roots the fold change was > 1.2, however, the *P*-value exceeded 0.05 so it was not included in microarray results. Nevertheless, it was additionally studied using RT-qPCR.

The ***CHLN*** gene was additionally included in the RT-qPCR study in leaves and roots due to the absence of corresponding probes on the GeneChip Tomato Genome Array

All accessions numbers are from NCBI.

**probe ID**	**probe hit**	**e value**	**L.4 vs WT Leaves**				**L.4 vs WT Roots**			
			**ESP**		**EPP**		**EC**		**S**	
			**p value**	**Fold change**	**p value**	**Fold change**	**p value**	**Fold change**	**p value**	**Fold change**
Les.3504.1.S1_at	AAD30548 iron-regulated transporter 1 *[Lycopersicon esculentum*]	2,00E-167	0.213255	1.01534	0.16075	-1.01756	0.233501	-1.17383	0.824129	1.02544
Les.4303.1.S1_at	AAP21818 root-specific metal transporter [*Lycopersicon esculentum*]	8,00E-276	0.281594	1.02867	0.185599	-1.03609	0.179653	-1.70218	0.473322	1.27078
Les.2567.1.S1_at	AAP21819 metal transporter *[Lycopersicon esculentum*]	4,00E-278					0.941705	-1.06408	0.785201	-1.22737
