# Supplementary material for: Engineering high Zn in tomato shoots through expression of AtHMA4 involves tissue-specific modification of endogenous genes
Source: BMC Genomics. 2016 Aug 12;17:625. doi: 10.1186/s12864-016-2990-x (PMC4982198; doi:10.1186/s12864-016-2990-x)
Supplement: Additional file 12: — Gene Ontology distribution of gene groups identified in leaves (PDF 570 kb) [file 12864_2016_2990_MOESM12_ESM.pdf]

Additional file 12: Gene Ontology distribution of the gene groups identified in leaves by the comparative microarray approach..

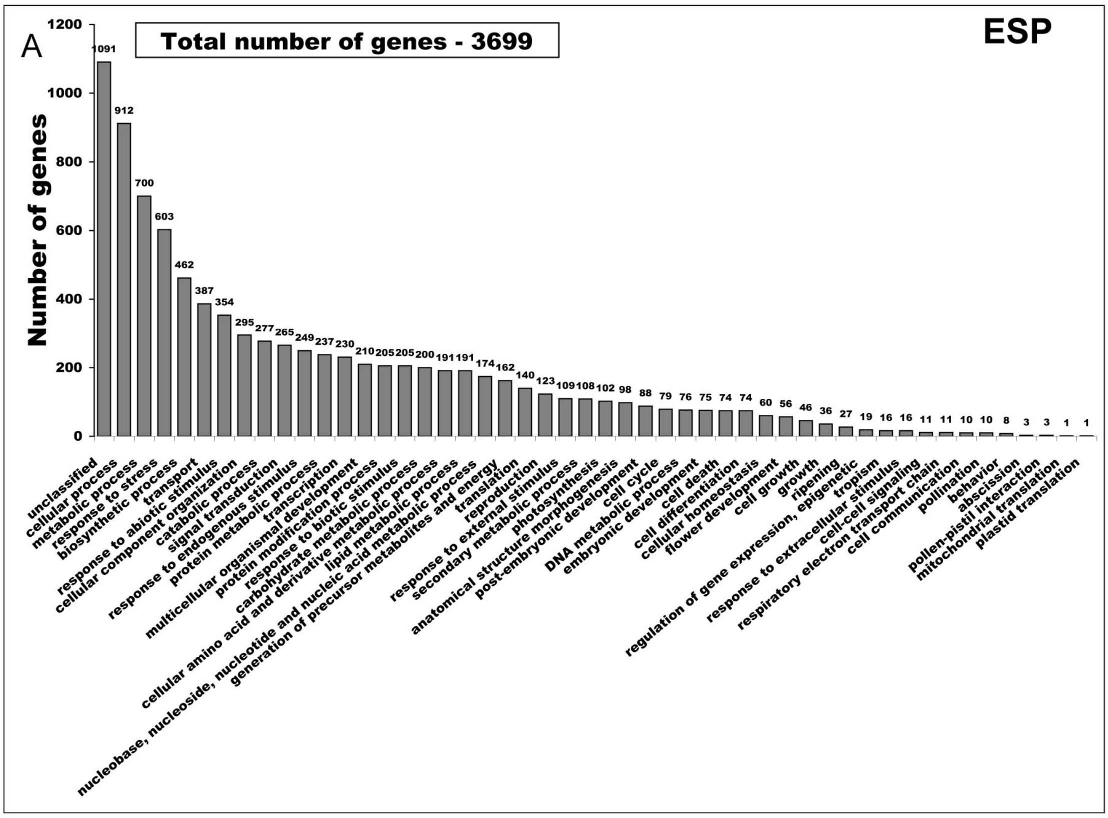

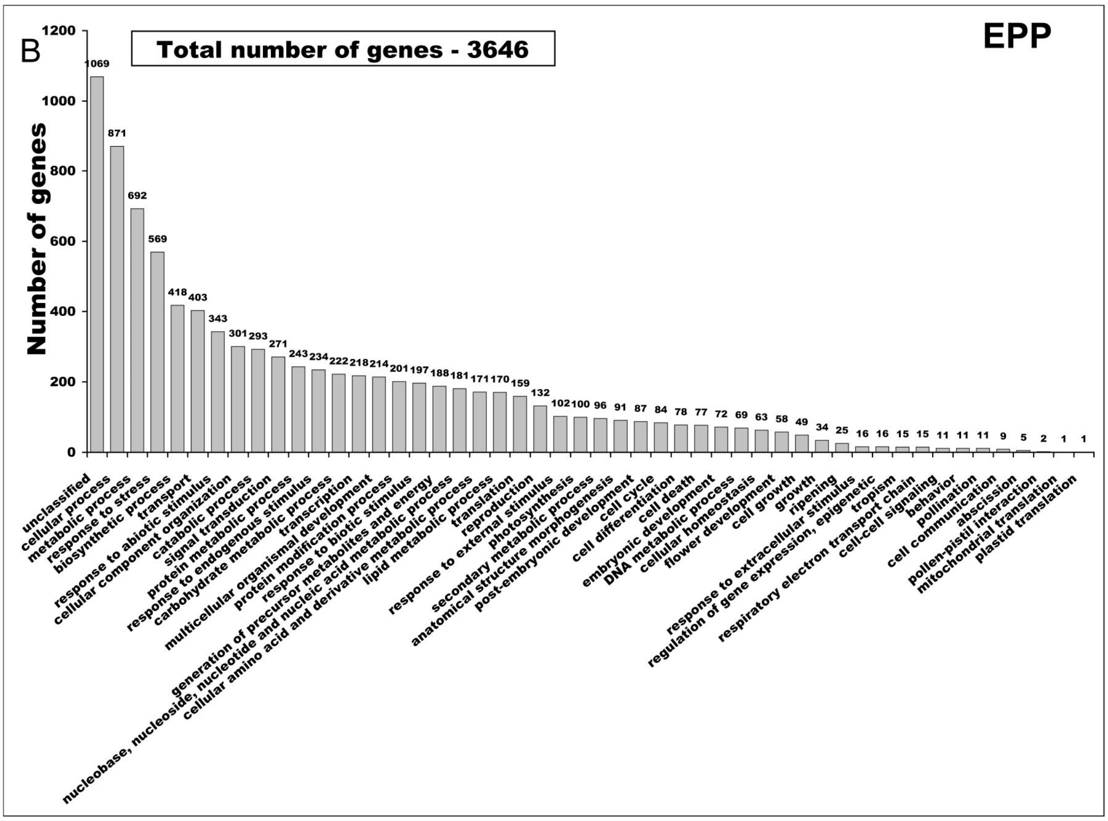

Genes were identified by microarray analysis as differentially expressed in (A) spongy parenchyma + lower epidermis and (B) palisade parenchyma + upper epidermis of leaves of

17-day-old *AtHMA4*-expressing tomato (line 4) as compared with the wild-type (WT) grown in the presence of 5 μM Zn for one week. Tomato genes were classified by GO of biological processes using the sequences from the Tomato Functional Genomics Database and GO tools available on the TFGD website.
