# Supplementary material for: Engineering high Zn in tomato shoots through expression of AtHMA4 involves tissue-specific modification of endogenous genes
Source: BMC Genomics. 2016 Aug 12;17:625. doi: 10.1186/s12864-016-2990-x (PMC4982198; doi:10.1186/s12864-016-2990-x)
Supplement: Additional file 13: — Alteration in transcript levels of selected genes identified in leaves by microarrays and verified by RT-qPCR. (PDF 21 kb) [file 12864_2016_2990_MOESM13_ESM.pdf]

Additional file 13: Alteration in transcript levels of selected genes identified in leaves by microarrays and verified by RT-qPCR. Changes in expression levels in lower epidermis+spongy paerenchyma (ESP) and upper epidermis+palisade paerenchyma (EPP) of leaves of 17-day old transgenic plants line 4 versus wild type Beta grown for one week in the presence of 5 µM Zn.

↑ - upregulated, ↓ - downregulated, "blank" - similar transcript level

In cases where transcripts were detected by Real-time PCR in one tissue type only, fold change could not be calculated. The results are then shown as arrows indicating the course of change.

All accessions numbers are from NCBI.

Accession nuber	Gene product	Microarray				RT-qPCR	
		ESP		EPP		ESP	EPP
		Fold change	p-value	Fold change	p-value	Fold change	Fold change
Metal transporters and uptake facilitators:							
AY562196	Natural resistance-associated macrophage protein 2 (*NRAMP2*)			↑1.3	0.0128031	↓	↑
AY196092	Natural resistance-associated macrophage protein 3 (*NRAMP3*)	↑ 1.2	0.000992745	↑ 1.2	0.000972334		↑
AY224079	Ferric-chelate reductase (*FRO1*)	↑ 1.5	$1.23958e^{-005}$	↑ 1.8	0.000162441	↓	↑
Transcription factors:							
FJ647190	Basic region/leucine zipper motif transcription factor 44 (*bZIP44)*	↑ 17.4	$1.32234e^{-011}$	↑ 11.3	$3.52978e^{-012}$	↑	↑
Ethylene pathway:							
NM_001247220	Ethylene receptor 1 (*ETR1*)			↑ 1.3	0.000651449	↓	↑
U38666	Never-ripe ethylene receptor (*NR*)	↑ 1.3	0.00391666	↑ 1.6	0.00014986	↓	↓
NM_001247095	1-Aminocyclopropane-1-carboxylate oxidase 1 (*ACO1*)	↑ 171.5	$3.99087e^{-011}$	↑ 165.7	$4.21099e^{-011}$	↓	↑
XM_004247652	1-Aminocyclopropane-1-carboxylate oxidase 3 (*ACO3, E8*)	↑ 2.1	0.00247568	↓ -1.7	0.000165337	↑	↓

NM_001246999	1-Aminocyclopropane-1-carboxylate oxidase 4 (*ACO4*)	↑ 5.6	$2.01684e^{-006}$	↑ 5.5	$2.1916e^{-006}$	↓	↑
NM_001247709	1-Aminocyclopropane-1-carboxylate oxidase 6 (*ACO6*)			↑ 1.5	$1.15738e^{-006}$	↓	↓
Cell wall modifications:							
U70677	Pectin methylesterase 1 (*PMEU1*)	↑ 1.3	$9.51282e^{-007}$	↑ 1.7	$2.85617e^{-009}$	↑	↑
NM_001246929	Xyloglucan endotransglucosylase/hydroxylase 1 (*XTH1*)	↓ -3.2	$2.13345e^{-007}$	↓ -2.7	$7.03154e^{-007}$	↓	↓
NM_001247543	Xyloglucan endotransglucosylase/hydroxylase 3 (*XTH3*)	↑ 12.0	$8.1219e^{-009}$	↑ 1,8	0.000299818		↑
NM_001245746	Xyloglucan endotransglucosylase/hydroxylase 7 (*XTH7*)	↓ -2.1	0.000310681	↓ -2.8	$2.92569e^{-005}$	↑	↓
U13055	endo-1,4-beta-D-glucanase (*Cel2*)			↓ -6.9	$4.99676e^{-006}$	↓	↓
U78526	endo-1,4-beta-D-glucanase (*Cel5*)	↓ -4.6	$2.86733e^{-006}$			↓	↑
AF308936	endo-1,4-beta-D-glucanase (*Cel7*)	↑ 25.1	$2.63431e^{-011}$	↑ 2.2	$1.72706e^{-006}$	↑	↑
