# Supplementary material for: Engineering high Zn in tomato shoots through expression of AtHMA4 involves tissue-specific modification of endogenous genes
Source: BMC Genomics. 2016 Aug 12;17:625. doi: 10.1186/s12864-016-2990-x (PMC4982198; doi:10.1186/s12864-016-2990-x)
Supplement: Additional file 14: — Alignments of LeNRAMP2 with NRAMPs from other species. (PDF 40 kb) [file 12864_2016_2990_MOESM14_ESM.pdf]

Additional file 14: Alignments of LeNRAMP2 with NRAMPs from other plant species

Part A

Aligment of predicted protein sequence LeNRAMP2 (XP_004238113.1, published in 2014, longer than AAS67887) with NRAMP2 proteins from *Arabidopsis thaliana* (AtNRAMP2 - NP_175157.1), *Oryza sativa* (OsNRAMP2 - AAB61961.1) and *Mus musculus* (mouse_*NRAMP2* - NP_001139633.1) Alignment was done using Clustal Omega program (http://www.ebi.ac.uk/Tools/msa/clustalo/). Residues were shaded with BoxShade (http://www.ch.embnet.org/software/BOX_form.html). Black and grey backgrounds indicate identical residues and conservative substitutions. Lines above the sequences indicate the positions of the transmembrane domains (TM). TM prediction program was used for prediction of transmembrane domain of LeNRAMP2 protein: (http://www.ch.embnet.org/software/TMPRED_form.html). The Consensus Transport Motif (CTM) between TMs 8 and 9 is boxed (according to Curie [21] –reference from the manuscript). Percent Identity Matrix created by Clustal2.1 is shown below alignment (rounded off to one decimal place). All accessions numbers are from NCBI.

```
LeNRAMP2        1 -MSSP-QQQENTSP--DSKEEESRHLLTAPLPQSTSPLINGDADDGEEEFVYGSGEKIHV
AtNRAMP2        1 --------MENDVK--ENLEEEEDRLLPPPPPSQSLPS-----TDSESEAAFETNEKILI
OsNRAMP2        1 ------------------------------------------------------------
mouse_NRAMP2    1 MVLDPKEKMPDDGASGDHGDSASLGAINPAYSNSSLPHSTG---DSEEPFTTYFDEKI--

                                                     TM I
LeNRAMP2       57 VEFDSVPIDGVDYSTVPPFSWKKLWQFTGPGFLMSIAFLDPGNLEGDLQAGAIAGYSLLW
AtNRAMP2       46 VDFES-PDDPTTGDTPPPFSWRKLWLFTGPGFLMSIAFLDPGNLEGDLQAGAIAGYSLLW
OsNRAMP2        1 --------------MRPAFSWRKLWRFTGPGFLMCIAFLDPGNLEGDLQAGAAAGYQLLW
mouse_NRAMP2   56 ------P---IPEEEYSCFSFRKLWAFTGPGFLMSIAYLDPGNIESDLQSGAVAGFKLLW

                      TM II                                   TM III
LeNRAMP2      117 LLLWATVMGLMIQLLSARIGVATGRHLAELCREEYPRWAGLLLWFMAEVALIGADIQEVI
AtNRAMP2      105 LLMWATAMGLLIQMLSARVGVATGRHLAELCRDEYPTWARYVLWSMAELALIGADIQEVI
OsNRAMP2       47 LLLWATVMGALVQLLSARLGVATGKHLAELCREEYPPWATAALWAMTELALVGADIQEVI
mouse_NRAMP2  107 VLLLATIVGLLLQRLAARLGVVTGLHLAEVCHRQYPKVPRIILWLMVELAIIGSDMQEVI

                                         TM IV                  TM V
LeNRAMP2      177 GSAIAIKILSRGVLPLWAGVLITASDCFLLLVLENYGIRKLEAVFAVLISTMALSFAWMF
AtNRAMP2      165 GSAIAIQILSRGFLPLWAGVVITASDCFLFLFLENYGVRKLEAVFAVLIATMGLSFAWMF
OsNRAMP2      107 GSAIAIKILSAGTVPLWGGVVITAFDCFIFLFLENYGVRKLEAFFGVLIAVMAVSFAIMF
mouse_NRAMP2  167 GSAIAINLLSAGRVPLWGGVLITIADTFVFLFLDKYGLRKLEAFFGFLITIMALTFGYEY

                                                     TM VI
LeNRAMP2      237 GDAKPNGKELLAGLLIPKLS---SRTVRQAVGVVGCVIMPHNVFLHSALVQSREIDLKKK
AtNRAMP2      225 GETKPSGKELMIGILLPRLS---SKTIRQAVGVVGCVIMPHNVFLHSALVQSRKIDPKRK
OsNRAMP2      167 GETKPSGKDLLIGLVVPKLS---SRTIKQAVGIVGCIIMPHNVFLHSALVESRKIDTNKK
mouse_NRAMP2  227 ITVKPSQSQVLRGMFVPSCPGCRTPQVEQAVGIVGAVIMPHNMYLHSALVKSRQVNRANK

                                     TM VII
LeNRAMP2      294 GQVQEALNYYSIESSFALLISFMINLFVTTVFAKGFYGSEQAGSLGLVNA----------
AtNRAMP2      282 SRVQEALNYYLIESSVALFISFMINLFVTTVFAKGFYGTEKANNIGLVNA----------
OsNRAMP2      224 SRVQEAVFYYNIESILALVVSFFINICVTTVFAKGFYGSEQADGIGLENA----------
mouse_NRAMP2  287 QEVREANKYFFIESCIALFVSFIINVFVVSVFAEAFFEKTNKQVVEVCKNNSSPHADLFP

                                    TM VIII
LeNRAMP2      344 ---GQFLQDKYGGGL-------FPILYIWGIGLLAAGQSSTITGTYAGQFIMGGFLDLRL
AtNRAMP2      332 ---GQYLQEKFGGGL-------LPILYIWGIGLLAAGQSSTITGTYAGQFIMGGFLNLRL
OsNRAMP2      274 ---GQYLQQKYGTAF-------FPILYIWAIGLLASGQSSTITGTYAGQFVMGGFLNLRL
mouse_NRAMP2  347 SDNSTLAVDIYKGGVVLGCYFGPAALYIWAVGILAAGQSSTMTGTYSGQFVMEGFLNLKW
```

```
                                TM IX                              TM X
LeNRAMP2      394 KKWLRALITRSCAIVPTIIVALIFNRSESSLDVLNEWLNVLQSIQIPFALIPLLTLVSKE
AtNRAMP2      382 KKWMRAVITRSCAIVPTMIVAIVFNTSEASLDVLNEWLNVLQSVQIPFALLPLLTLVSKE
OsNRAMP2      324 KKWLRAMITRSFAIIPTMIVALFFDTEDPTMDILNEALNVLQSIQIPFALIPLITLVSKE
mouse_NRAMP2  407 SRFARVILTRSIAIIPTLLVAVFQDVE--HLTGMNDFLNVLQSLQLPFALIPILTFTSLR

                                         TM XI                        TM XII
LeNRAMP2      454 DIMGTFKIGPALERVAWTIAVLVMVINGYLLLDFFVSEVNGPLFAFLVCAGTAGYVAFIL
AtNRAMP2      442 EIMGDFKIGPILQRIAWTVAALVMIINGYLLLDFFVSEVDGFLFGVTVCVWTTAYIAFIV
OsNRAMP2      384 QVMGSFVVGPITKVISWIVTVFLMLINGYLILSFYATEVRGALVRSSLCVVLAVYLAFIV
mouse_NRAMP2  465 PVMSEFSNGIGWRIAGGILVLIVCSINMYFVVV-YVQELGHVALYVVAAVVSVAYLTFVF

LeNRAMP2      514 YLISHGGGNVANWFNLLRTKG--YSYAGQ------------
AtNRAMP2      502 YLISHSNFFPSPWSSSSIELPKRVSVSNS------------
OsNRAMP2      444 YLIMQNTSLYSRLRSAMTKST--------------------
mouse_NRAMP2  524 YLGWQCLIALG---LSFLDCGRSVSISKVLLSEDTSGGNIK
```

	1	2	3	4
1: LeNRAMP2	100.0	74.2	68.7	44.6
2: AtNRAMP2	74.2	100.0	70.3	46.2
3: OsNRAMP2	68.7	70.3	100.0	50.0
4: mouse_NRAMP2	44.6	46.2	50.0	100.0

Part B

Alignment of predicted protein sequence LeNRAMP2 (XP_004238113.1, published in 2014, longer than AAS67887) with NRAMPs proteins from *Arabidopsis thaliana* (AtNRAMP1- NP_178198.1, AtNRAMP2- NP_175157.1, AtNRAMP3 - NP_179896.1, AtNRAMP4 - NP_201534.1, AtNRAMP5 - NP_193614.1, AtNRAMP6 - NP_173048.3, and *Noccaea caerulescens* (formerly *Thlaspi caerulescens*, TcNRAPM3 - ABR27746.1, TcNRAMP4 - ABR27747.1) Alignment was done using Clustal Omega program. Residues were shaded with BoxShade (http://www.ch.embnet.org/software/BOX_form.html). Black and grey backgrounds indicate identical residues and conservative substitutions. Lines above the sequences indicate the positions of the transmembrane domains. TM prediction program was used (http://www.ch.embnet.org/software/TMPRED_form.html) for prediction of transmembrane domain of LeNRAMP2 protein. The Consensus Transport Motif (CTM) between TMs 8 and 9 is boxed (according to Curie [21] –reference from the manuscript) Percent Identity Matrix created by Clustal2.1 is shown below alignment (rounded off to one decimal place)
All accessions numbers are from NCBI.

```
LeNRAMP2     1 ----MSSPQQQENTSPDSKEEESRHLLTAPLPQSTSPLINGDADDGEEEFVYGSGEKIHV
AtNRAMP2     1 ----------MENDVKENLEEEEDRLLPPP-----PPSQSLPSTDSESEAAFETNEKILI
AtNRAMP3     1 -----------------MPQLENNE-----------PLLINE--EEEEETAYDETEKVHI
TcNRAMP3     1 -----------------MSRLENDR-----------PLLIDRIDEEEEETAYDETEKVHI
TcNRAMP4     1 -----------------MSETERER-----------PLLA------SEERAYEETEKVHI
AtNRAMP4     1 -----------------MSETDRER-----------PLLA------SEERAYEETEKVLI
AtNRAMP5     1 MTGSTVSRQENSPKRPNDSNGEFKRLLVPETSQ------------PEEDELHESPPENQI
AtNRAMP6     1 ---------------------------------------------MAAETASGSNRSI
AtNRAMP1     1 -----------------MAATGSGR----------------------SQFISSSGGNRSF
```

```
                                                            TM I

LeNRAMP2   57 VEFDSVPIDGVDY----STVPPFSWKKLWQFTGPGFLMSIAFLDPGNLEGDLQAGAIAGY
AtNRAMP2   46 VDFESPDDP-----TTGDTPPPFSWRKLWLFTGPGFLMSIAFLDPGNLEGDLQAGAIAGY
AtNRAMP3   31 VRNEE--EDDLEHGVGCGGAPPFSWKKLWLFTGPGFLMSIAFLDPGNLEGDLQAGAVAGY
TcNRAMP3   33 VRDEDDNERDLEYGVGCGGAPPFSWRKLWLFTGPGFLMSIAFLDPGNLEGDLQSGAVAGY
TcNRAMP4   27 VGVDE--EDDANYD-ELGNAPRFSWKKLWLFTGPGFLMSIAFLDPGNLESDLQAGAIAGY
AtNRAMP4   27 VGIDE--EEDADYDDDPGNSPKFSWKKLWLFTGPGFLMSIAFLDPGNLESDLQAGAIAGY
AtNRAMP5   49 LNVEE------DRDKTYDSVPPFSWAKLWKFTGPGFLMSIAFLDPGNIEGDLQAGAVAGY
AtNRAMP6   14 S--NSPLIEN-SDSNQILVPEKKSWKNFFSYLGPGFLVSIAYIDPGNFETDLQSGAQYKY
AtNRAMP1   22 S--NSPLIEN-SDSNQIIVSEKKSWKNFFAYLGPGFLVSIAYIDPGNFETDLQAGAHYKY

                  TM II                                  TM III

LeNRAMP2  113 SLLWLLLWATVMGLMIQLLSARIGVATGRHLAELCREEYPRWAGLLLWFMAEVALIGADI
AtNRAMP2  101 SLLWLLMWATAMGLLIQMLSARVGVATGRHLAELCRDEYPTWARYVLWSMAELALIGADI
AtNRAMP3   89 SLLWLLMWATAMGLLVQLLSARLGVATGRHLAELCRDEYPTWARMVLWVMAELALIGSDI
TcNRAMP3   93 SLLWLLMWATAMGLLVQLLSARLGVATGRHLAELCREEYPSWAGMVLWVMAELALIGSDI
TcNRAMP4   84 SLIWLLMWATAIGLLIQLLSARLGVATGRHLAELCREEYPTWARMVLWIMAEIALIGADI
AtNRAMP4   85 SLIWLLMWATAIGLLIQLLSARLGVATGRHLAELCREEYPTWARMVLWIMAEIALIGADI
AtNRAMP5  103 SLLWLLLWATLMGLLMQLLSARIGVATGRHLAEICRSEYPSWARILLWFMAEVALIGADI
AtNRAMP6   71 ELLWIILVASCAALVIQSLAANLGVVTGKHLAEHCRAEYSKVPNFMLWVVAEIAVVACDI
AtNRAMP1   79 ELLWIILVASCAALVIQSLAANLGVVTGKHLAEQCRAEYSKVPNFMLWVVAEIAVVACDI

                                           TM IV               TM V

LeNRAMP2  173 QEVIGSAIAIKILSRGVLPLWAGVLITASDCFLLLVLENYGIRKLEAVFAVLISTMALSF
AtNRAMP2  161 QEVIGSAIAIQILSRGFLPLWAGVVITASDCFLFLFLENYGVRKLEAVFAVLIATMGLSF
AtNRAMP3  149 QEVIGSAIAIKILSNGILPLWAGVVITALDCFVFLFLENYGIRKLEAVFAVLIATMGVSF
TcNRAMP3  153 QEVIGSAIAIKILTNGILPLWAGVIITALDCFFFLFFENYGIRKLEAVFAVLIATMGVSF
TcNRAMP4  144 QEVIGSAIAIKILSNGLIPLWAGVVITALDCFIFLFLENYGIRKLEAVFAILIATMAVAF
AtNRAMP4  145 QEVIGSAIAIKILSNGLVPLWAGVVITALDCFIFLFLENYGIRKLEAVFAILIATMALAF
AtNRAMP5  163 QEVIGSAIALQILTRGFLPIWVGVIITSFDCFLISYLEKCGMRKLEGLFAVLIATMALSF
AtNRAMP6  131 PEVIGTAFALNMLFN--IPVWIGVLLTGLSTLILLALQQYGIRKLEFLIAFLVFTIALCF
AtNRAMP1  139 PEVIGTAFALNMLFS--IPVWIGVLLTGLSTLILLALQKYGVRKLEFLIAFLVFTIAICF

                                            TM VI

LeNRAMP2  233 AWMFGDAKPNGKELLAGLLIPKLSSRT-VRQAVGVVGCVIMPHNVFLHSALVQSREIDLK
AtNRAMP2  221 AWMFGETKPSGKELMIGILLPRLSSKT-IRQAVGVVGCVIMPHNVFLHSALVQSRKIDPK
AtNRAMP3  209 AWMFGQAKPSGSELLIGILVPKLSSRT-IQKAVGVVGCIIMPHNVFLHSALVQSREVDKR
TcNRAMP3  213 AWMFGQAKPSGSELLVGILVPKLSSRT-IQKAVGVVGCIIMPHNVFLHSALVQSREVDKR
TcNRAMP4  204 AWMFGQTKPSGTELLVGALVPKLSSRT-IKQAVGIVGCIIMPHNVFLHSALVQSREVDPK
AtNRAMP4  205 AWMFGQTKPSGTELLVGALVPKLSSRT-IKQAVGIVGCIIMPHNVFLHSALVQSREVDPK
AtNRAMP5  223 AWMFNETKPSVEELFIGIIIPKLGSKT-IREAVGVVGCVITPHNVFLHSALVQSRKTDPK
AtNRAMP6  189 FVELHYSKPDPKEVLYGLFVPQLKGNGATGLAISLLGAMVMPHNLFLHSALVLSRKIP-R
AtNRAMP1  197 FVELHYSKPDPGEVLHGLFVPQLKGNGATGLAISLLGAMVMPHNLFLHSALVLSRKIP-R

                                  TM VII

LeNRAMP2  292 KKGQVQEALNYYSIESSFALLISFMINLFVTTVFAKGFYGSEQ-------AGSLGLVNAG
AtNRAMP2  280 RKSRVQEALNYYLIESSVALFISFMINLFVTTVFAKGFYGTEK-------ANNIGLVNAG
AtNRAMP3  268 QKYRVQEALNYYTIESTIALFISFLINLFVTTVFAKGFYNTDL-------ANSIGLVNAG
TcNRAMP3  272 QKYRVQEAINYYTIESTLALFVSFLINLFVTTVFAKGFYNTDL-------ADSIGLVNAG
TcNRAMP4  263 NRFRVKEALKYYSIESAGALAVSFIINVFVTTVFAKSFYGTDI-------AETIGLANAG
AtNRAMP4  264 KRFRVKEALKYYSIESTGALAVSFIINVFVTTVFAKSFYGTEI-------ADTIGLANAG
AtNRAMP5  282 EINRVQEALNYYTIESSAALFVSFMINLFVTAVFAKGFYGTKQ-------ADSIGLVNAG
AtNRAMP6  248 SVTGIKEACRYYLIESGLALMVAFLINVSVISVSGAVCNASDLSPEDRASCQDLDLNKAS
AtNRAMP1  256 SASGIKEACRFYLIESGLALMVAFLINVSVISVSGAVCNAPNLSPEDRANCEDLDLNKAS
```

```
                                 TM VIII
LeNRAMP2  345 QFLQDKYGGGLFPILYIWGIGLLAAGQSSTITGTYAGQFIMGGFLDLRLKKWLRALITRS
AtNRAMP2  333 QYLQEKFGGGLLPILYIWGIGLLAAGQSSTITGTYAGQFIMGGFLNLRLKKWMRAVITRS
AtNRAMP3  321 QYLQEKYGGGVFPILYIWAIGLLAAGQSSTITGTYAGQFIMGGFLNFKMKKWLRALITRS
TcNRAMP3  325 QYLQDKYGGGLFPILYIWGIGLLAAGQSSTITGTYAGQFIMGGFLNFRMKKWMRALITRS
TcNRAMP4  316 QYLQDKYGRGYFPILYIWAIGVLAAGQSSTITGTYAGQFIMGGFLNLKMKKWVRALITRS
AtNRAMP4  317 QYLQDKYGGGFFPILYIWAIGVLAAGQSSTITGTYAGQFIMGGFLNLKMKKWVRALITRS
AtNRAMP5  335 YYLQEKYGGGVFPILYIWGIGLLAAGQSSTITGTYAGQFIMEGFLDLQMEQWLSAFITRS
AtNRAMP6  308 FLLRNVV-GKWS--SKLFAIALLASGQSSTITGTYAGQYVMQGFLDLRLEPWLRNFLTRC
AtNRAMP1  316 FLLRNVV-GKWS--SKLFAIALLASGQSSTITGTYAGQYVMQGFLDLRLEPWLRNLLTRC

                    TM IX                   TM X
LeNRAMP2  405 CAIVPTIIVALIFNRSESSLDVLNEWLNVLQSIQIPFALIPLLTLVSKEDIMGTFKIGPA
AtNRAMP2  393 CAIVPTMIVAIVFNTSEASLDVLNEWLNVLQSVQIPFALLPLLTLVSKEEIMGDFKIGPI
AtNRAMP3  381 CAIIPTIIVALVFDSSEATLDVLNEWLNVLQSIQIPFALIPLLCLVSKEQIMGSFKIGPL
TcNRAMP3  385 CAIIPTIIVALVFDSSEATLDVLNEWLNVLQSIQIPFALIPLLCLVSKERIMGSFKIGPL
TcNRAMP4  376 CAIIPTMIVALVFDSSASMLDELNEWLNVLQSVQIPFAVIPLLCLVSNEQIMGSFKIQPL
AtNRAMP4  377 CAIIPTMIVALVFDSSDSMLDELNEWLNVLQSVQIPFAVIPLLCLVSNEQIMGSFKIQPL
AtNRAMP5  395 FAIVPTMFVAIMFNTSEGSLDVLNEWLNILQSMQIPFAVIPLLTMVSNEHIMGVFKIGPS
AtNRAMP6  365 LAIIPSLIVALIGGSAGAGK--LIIIASMILSFELPFALVPLLKFTSSKTKMGSHANSLV
AtNRAMP1  373 LAIIPSLIVALIGGSAGAGK--LIIIASMILSFELPFALVPLLKFTSCKTKMGSHVNPMA

                                                  TM XII
                      TM XI
LeNRAMP2  465 LERVAWTIAVLVMVINGYLLLDFFVSEVNG--------PLFAFLVCAGTAGYVAFILYLI
AtNRAMP2  453 LQRIAWTVAALVMIINGYLLLDFFVSEVDG--------FLFGVTVCVWTTAYIAFIVYLI
AtNRAMP3  441 YKTIAWLVAALVIMINGYLLLEFFSNEVSG--------IVYTGFVTLFTASYGAFILYLI
TcNRAMP3  445 CQTIAWLVAALVIMINGYLLIEFFSSEVSG--------IVYTGFVTVFTALYGAFIVYLI
TcNRAMP4  436 VQTISWIVAALVISINGYLMVDFFSGAAKS--------VVLLVLVIIFAVAYVLFVLYLI
AtNRAMP4  437 VQTISWIVAALVIAINGYLMVDFFSGAATN--------LILLVPVIIFAIAYVVFVLYLI
AtNRAMP5  455 LEKLAWTVAVFVMMINGYLLLDFFMAEVEG--------FLVGFLVFGGVVGYISFIIYLV
AtNRAMP6  423 ISSVTWIIGGLIMGINIYYLVSSFIKLLLHSHMNLVAIVFLGVLGFSGIATYLAAISYLV
AtNRAMP1  431 ITALTWVIGGLIMGINIYYLVSSFIKLLIHSHMKLILVVFCGILGFAGIALYLAAIAYLV

LeNRAMP2  517 SHGGGNVANWFNLLRTKG----YSYAGQ-----------------
AtNRAMP2  505 SHSNFFPSPWSSS--SIE----LPKRVSVSNS-------------
AtNRAMP3  493 ARGITF-TPWPFKAESSH---------------------------
TcNRAMP3  497 ARGINF-TPWRPKAESS----------------------------
TcNRAMP4  488 SRGLTY-TPCN----------------------------------
AtNRAMP4  489 SRGLTY-TPWQLVASSHK----EPQRDDE----------------
AtNRAMP5  507 SYRSSQSSSWSSLEMSER----VVSTET-----------------
AtNRAMP6  483 LRKNRESSSTHFLDFSNSQTEETLPREDIANMQLPNRVAVIGDLN
AtNRAMP1  491 FRKNRVATS--LLISRDSQNVETLPRQDIVNMQLPCRVSTSDVD-
```

	1	2	3	4	5	6	7	8	9
1: LeNRAMP2	100.0	74.2	72.1	71.5	69.6	68.2	67.6	40.7	40.2
2: AtNRAMP2	74.2	100.0	74.7	73.9	70.9	70.5	70.3	38.7	37.4
3: AtNRAMP3	72.1	74.7	100.0	92.1	78.1	76.4	66.1	39.5	38.4
4: TcNRAMP3	71.5	73.7	92.1	100.0	76.7	75.6	65.8	39.2	38.3
5: TcNRAMP4	69.6	70.9	78.1	76.7	100.0	94.4	63.4	40.0	39.2
6: AtNRAMP4	68.2	70.5	76.4	75.6	94.4	100.0	62.0	39.3	38.9
7: AtNRAMP5	67.6	70.3	66.1	65.8	63.4	62.0	100.0	37.9	36.7
8: AtNRAMP6	40.7	38.7	39.5	39.2	40.0	39.3	37.9	100.0	87.2
9: AtNRAMP1	40.2	37.4	38.4	38.3	39.2	38.9	36.7	87.2	100.0
